# CSN: unsupervised approach for inferring biological networks based on the genome alone

**DOI:** 10.1186/s12859-020-3479-9

**Published:** 2020-05-15

**Authors:** Maya Galili, Tamir Tuller

**Affiliations:** 1 Biomedical Engineering Department, Tel Aviv University, Tel-Aviv, Israel; 2 Department of Molecular Microbiology & Biotechnology, Tel Aviv University, Tel-Aviv, Israel; 3 The Sagol School of Neuroscience, Tel Aviv University, Tel-Aviv, Israel

**Keywords:** Biological networks, Transcripts comparison, Gene function annotation, Gene expression, *S. cerevisiae*, *E. coli*

## Abstract

**Background:**

Most organisms cannot be cultivated, as they live in unique ecological conditions that cannot be mimicked in the lab. Understanding the functionality of those organisms’ genes and their interactions by performing large-scale measurements of transcription levels, protein-protein interactions or metabolism, is extremely difficult and, in some cases, impossible. Thus, efficient algorithms for deciphering genome functionality based only on the genomic sequences with no other experimental measurements are needed.

**Results:**

In this study, we describe a novel algorithm that infers gene networks that we name Common Substring Network (CSN). The algorithm enables inferring novel regulatory relations among genes based only on the genomic sequence of a given organism and partial homolog/ortholog-based functional annotation. It can specifically infer the functional annotation of genes with unknown homology.

This approach is based on the assumption that related genes, not necessarily homologs, tend to share sub-sequences, which may be related to common regulatory mechanisms, similar functionality of encoded proteins, common evolutionary history, and more.

We demonstrate that CSNs, which are based on *S. cerevisiae* and *E. coli* genomes, have properties similar to ‘traditional’ biological networks inferred from experiments. Highly expressed genes tend to have higher degree nodes in the CSN, genes with similar protein functionality tend to be closer, and the CSN graph exhibits a power-law degree distribution. Also, we show how the CSN can be used for predicting gene interactions and functions.

**Conclusions:**

The reported results suggest that ‘silent’ code inside the transcript can help to predict central features of biological networks and gene function. This approach can help researchers to understand the genome of novel microorganisms, analyze metagenomic data, and can help to decipher new gene functions.

**Availability:**

Our MATLAB implementation of CSN is available at https://www.cs.tau.ac.il/~tamirtul/CSN-Autogen

## Background

In recent years, technologies and tools for new organisms’ genomes sequencing are improving at an exponential rate [1, 2]. Today, there are over 150 K full genomes [3] from the Zika virus [4] to Giraffe [5], including various sets of metagenomics data [6]. Various biological and computational approaches have been developed for determining the coding regions [7–9]. Deciphering the function of genes, interactions between genes, relations between genotype and phenotype, and genome complexity. However, it is still a very challenging mission with only partial success (see, for example, [10–17]. Only in a small number of well-studied model organisms, various experimental tools such as gene expression measurements [18, 19], protein-protein interactions [PPI] measurements [20–22], genetic interaction measurements [23–25], and others, have been combined to decipher the functionality of genes and the way they work together. Even for those model organisms there are still many open questions regarding the exact functionality of genes [16, 26–28], and for most organisms, these data is still limited (see, for example, [29–32]. This fact makes the research in related topics very challenging [33–37]. Some computational tools have been tried to solve the protein function prediction challenge by integrating high-end algorithms and annotated data [38, 39]. The conventional approach is based on the homology of proteins, but it cannot be implemented for deciphering the functionality of novel genes with no well-studied homologs.

In this study, we propose a generic approach that generates comprehensive networks of interactions/similarity among genes based only on the organism’s genome. This method can help to predict gene functions, the interaction between genes, and gene expression levels. The approach is based, among others, on a measure that exploits in an unsupervised manner various gene expression codes which are interleaved in the coding region or the promoter, in addition to the protein functionality which is encoded in proteins’ amino acid content [16, 40–46].

By implementing our algorithm on all *S. cerevisiae* and *E. coli* genes, and generating the complete genomic map, which is based solely on its Deoxyribonucleic Acid [DNA] sequence, we demonstrate that our approach gives meaningful predictions. Moreover, we show that those predictions are comparables to the ones provided by experimental-based biological networks. We also show how our approach can be used for analyzing metagenomic samples. The results for the tested cases show that the CSN can reveal information regardless of the source domain of the genes, genome size, or sequences length.

## Results

### Developing a comprehensive sequence-based network - the CSN

**Fig. 1:**
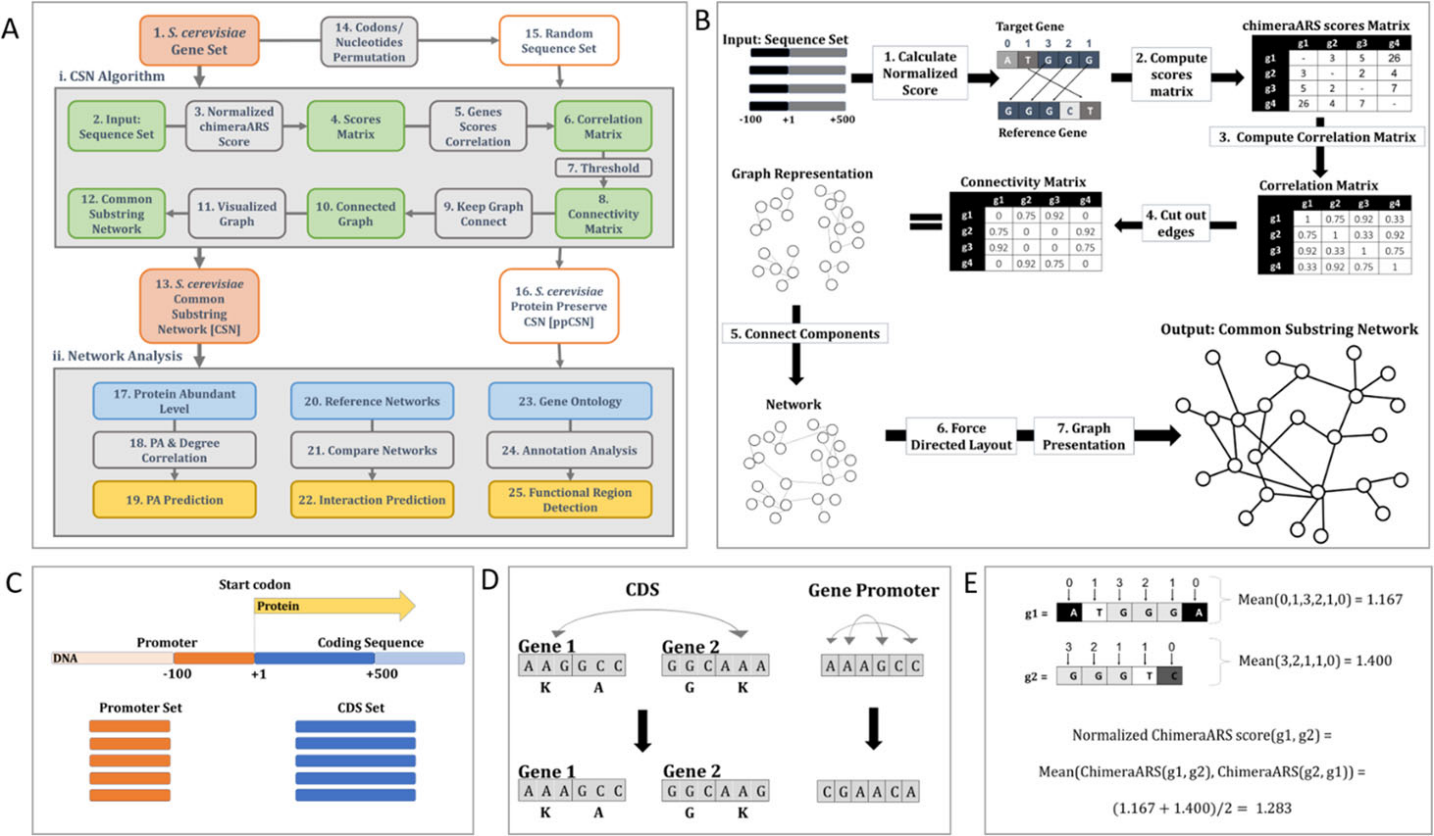
The framework of the Common Substring Network algorithm. **a** Flow diagram of the research, including the data, algorithms, and analyses. Box 1 & box 15: input data. Box 14: randomization method. Frame i: Algorithm’s steps. Box 13 & box 16: output networks. Frame ii: Analysis methods. See more details in the main text. **b** Graphical illustration of the CSN algorithm’s major steps - (details in the main text). **c** The different types of sequences in the *S. cerevisiae* genome. Promoter sequence (orange) and CDS sequence (blue) are treated separately and for that are organized in two different sets. **d** Different randomization methods for CDS and UTRs. A different randomization method was performed for each part of the gene: In the case of CDS, each set of synonymous codons was shuffled among all analyzed sequences (left). In the case of promoter sequences, we permuted the nucleotide order of each sequence (right). **e** Normalized chimeraARS score calculation example

The CSN network is constructed by calculating the resemblance scores between all pairs of genes based on their nucleotide sequences (Fig. 1a, box (i). All details appear in the Methods section). The CSN algorithm input is a set of sequences (Fig. 1a, box 2); in the first step, a unique distance measure which is called Normalized chimera Average Repetitive Substring (chimeraARS) is calculated for all pairs of genes (Fig.1a, box 3; Fig. 1b, step 1).

All these scores are organized in a symmetric similarity matrix (Fig.1a, box 4; Fig. 1b, step 2). In the second step, correlations coefficients between rows in the matrix mentioned above are calculated (Fig. 1a, box 5; Fig. 1b, step 3), and arranged in a new, correlation-based, similarity matrix (Fig. 1a, box 6): each entry is related to a pair of genes, and higher values are related to higher similarity. From this similarity matrix, an undirected-weighted graph can be induced. In this graph, genes with higher similarity are connected by an edge with higher weight. By filtering out low weights’ edges related to low similarities (Fig. 1a, boxes 7 & 8; Fig. 1b, step 4), and by adding a minimal number of edges (Fig. 1a, boxes 9 & 10; Fig. 1b, step 5), we make sure that the graph remains connected. A force-directed network layout algorithm (Fig. 1a, box 11; Fig. 1b, step 6) is applied to get the two dimensional (2D) network we call CSN (Fig. 1a, box 12); thus, the CSN can be easily visualized with a network visualization tool (Fig. 1a, box 11; Fig. 1b, step 7).

We aimed at evaluating the CSN’s ability to “capture” and exploit complex regulatory information, which is encoded in the ‘silent’ aspects of genes, and to assess the importance of this type of information. Specifically, most of the previous methods in the field for functionality estimation consider only the amino acid content of proteins; thus, we estimating the amount of information they may miss. To this end, we took the same initial set of sequences as the CSN; but in this CSN input, we shuffled the nucleotide sequence in a way that preserves the encoded protein but changes the codons’ order (Fig. 1a, box 14). By applying the same pipeline for generating the CSN but on these randomized sequences, we created a CSN analogous network that we called ppCSN - protein preserving Common Substring Network (Fig. 1a, box 16). The ppCSN is based on “randomized” sequences (Fig. 1a, box 15) which maintain the amino acid sequence of the input to the CSN’s sequences. Moreover, all the main gene features (e.g., the frequencies of nucleotides and codons, and GC content) are also preserved. Thus, the exact DNA sequences (see Methods section Randomization & Validation to more details) are modified.

### Inferring the CSN of *S. cerevisiae* and *E. coli*

**Fig. 2:**
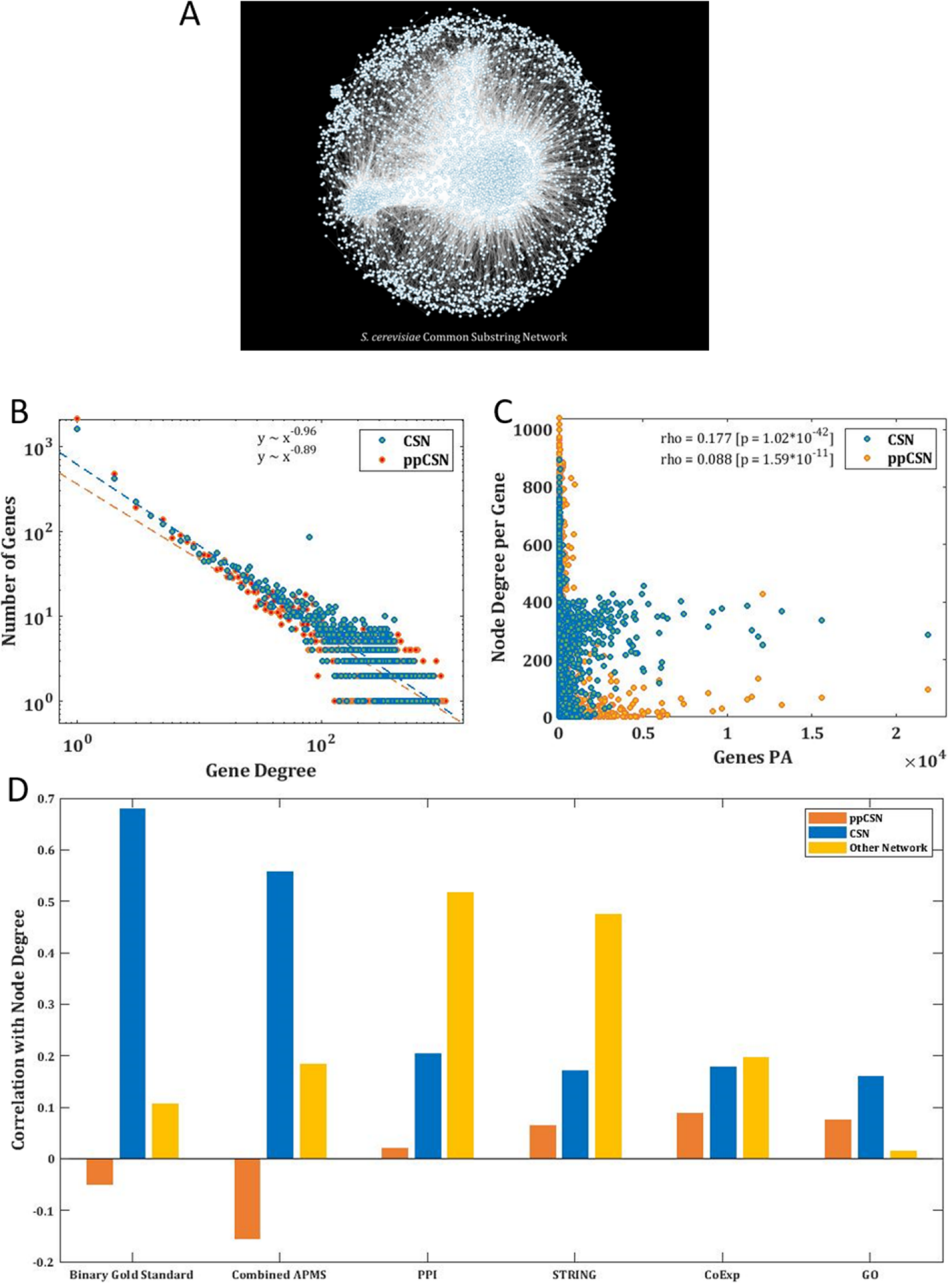
CSN Basic Properties. **a** The Common Substring Network of *S. cerevisiae*. This network represents 5972 genes and 237,237 edges. **b** The distribution of node degrees. Dot plot describing the distribution of nodes’ degrees in a log-log scale for the CSN’s nodes (blue) and the ppCSN’s nodes (orange). The dashed line represents the regression lines between node degree and number of times it appears in the graph. [Pearson: CSN rho = − 0.167 *p* = 8.78*10^− 5^, ppCSN rho = − 0.129 *p* = 1.05*10^− 3^; Spearman: CSN rho = − 0.838, *p* = 2.90*10^− 145^, ppCSN rho = − 0.759 *p* = 1.97*10^− 121^] **c** Spearman correlation between node degree and PA. Dot plot describing the linear regression relationship between gene degrees and their PA for CSN (blue) and the ppCSN (orange). [Spearman: CSN rho = 0.177; *p* = 1.02*10^− 42^, ppCSN rho = 0.088; *p* = 1.59*10^− 11^; Pearson: CSN rho = 0.219; *p* = 1.51*10^− 64^, ppCSN rho = − 0.027; *p* = 4.08*10^− 2^] **d** Comparison of PA and degree correlation. Between CSN (blue), ppCSN (orange), and other biological networks (see the supplementary section, Reference networks, yellow) PA and degree correlation

Our aim in the rest of this study was to show that the CSN can be used to exploit meaningful information related to gene function, gene interactions, and gene expression, which is compatible with the information provided by networks generated based on experiments. Thus, to evaluate our approach, we applied it on highly studied organisms from two different life domains: the baker’s yeast (*S. cerevisiae*), as a representative of eukaryotes (Fig. 1a, box 1) and *E. coli* as a prokaryotic representative; we also applied our algorithm on metagenomic sample *MGYA00382686* as an example. We carried out a thorough examination of the resultant full gene set CSN graphs (Fig. 1a, box 13) and its properties (see Fig. 1a. box (ii)). First, we examined CSN ability to predict protein abundance [PA], based on the CSN nodes’ centrality (Fig. 1a, boxes 17–19). The results were compared to ppCSN (Fig. 1A, box 16) and to others, well-establish biological networks, which are based on experimental data. Next, we compared the edges in CSN to interactions that appear in biological networks based on experiments (Fig. 1a, boxes 20–21) to determine its ability to predict various interactions between genes and their products (Fig. 1a, box 22). Finally, based on the functional annotation of genes (Fig. 1a, box 23), we performed a novel clustering procedure on the CSN nodes (Fig. 1a, box 24) to show that the CSN’s nodes are arranged according to their functionality (Fig. 1a, box 25). Figure 2a displays the CSN for *S. cerevisiae* ‘s genome.

### CSN degree distribution enable protein expression prediction

We found that the CSN nodes’ degrees are presenting power-law distribution, which is known as a fundamental feature of biological networks [16, 47, 48]. Specifically, it has been suggested that the distribution of degree in biological networks tends to be scale-free with a linear relation in a log-log graph for nodes’ degree distributiont [16, 48]. Examining CSN’s nodes’ degree distribution in linear fit shows indeed a significant negative correlation between node degree and frequency (Pearson: rho = − 0.167; *p* = 8.78*10^− 5^; Spearman: rho = − 0.838; *p* = 2.90*10^− 145^) which is stronger than the correlation obtained for the ppCSN graph (Pearson: rho − 0.129; *p* = 1.05*10^− 3^; Spearman: rho = − 0.759; *p* = 1.97*10^− 121^) (Fig. 2b). The analysis has yielded significant correlations on the *E. coli* CSN’s graph (Pearson: rho = − 0.363; *p* = 3.41*10^− 12^; Spearman: rho = − 0.913 *p* = 2.26*10^− 135^) and for its ppCSN (Pearson: rho = − 0.408; *p* = 3.64*10^− 16^; Spearman: rho = − 0.897 *p* = 1.65*10^− 131^) (Fig. S12E).

The node centrality of biological networks can correlate with various fundamental measures of gene/protein [47, 49, 50]. In many cases, hubs, which are usually central genes, tend to hold an important regulatory role, be essential to the cell or show high expression level [51, 52] (Fig. S10). The Spearman correlation between CSN nodes’ degrees and their PA [53], is significant but not very high (Pearson: rho = 0.219 *p* = 1.51*10^− 64^; Spearman: rho = 0.177; *p* = 10^− 42^), this supports the conjecture that there is a weak monotone relation between the two variables (Fig. 2c). The same analysis was conduct on *E. coli’s* CSN/ppCSN graphs (Pearson: rho = 0.023; *p* = 0.134; Spearman: rho = 0.151; *p* = 3.01*10^− 22^), here also, the correlation is stronger than the correlation obtained for the ppCSN graph (Pearson: rho = 0.011; *p* = 0.498; Spearman: rho = 0.094; *p* = 1.99*10^− 9^) (Fig. S12G).

Since the ppCSN node degrees correlation with their PA ‘s is significantly lower than the CSN’s correlation on both organisms; (Spearman: rho = 0.088; *p* = 1.59*10^− 11^; Pearson: rho = − 0.027 *p* = 4.08*10^− 2^) for *S. cerevisiae* CSN, and (Spearman: rho = 0.094; p = 1.99*10^− 9^; Pearson: rho = 0.011; *p* = 0.4.98) for *E. coli’s* ppCSN, we conclude that this association is mainly related to complex ‘silent’ aspects of the genes and not only to its amino acid content (Fig. 2c), or the transcript’s basic features such as codon frequencies and GC content.

Interestingly, we were able to improve the PA predictive power of CAI, a feature that is usually used as protein abundance predictor [54], by adding the CSN degree as an additional feature. Specifically, the correlation with PA of a regressor, which is based on CAI and the CSN node’s degree, is higher than the correlation between PA and CAI (Supplementary, Fig. S14). When comparing the expression prediction by degree against six different existing genetic networks assembly methods, some of them aggregate many techniques and a data source such as STRING [20], CSN exhibits the best correlation between nodes degree and PA (Fig. 2d). The comparison here is carefully considering the common gene list and the general network density to make sure that the degree correlations are not biased (see supplementary Data Preprocessing section).

### CSN has predictive power of gene interactions

**Fig. 3:**
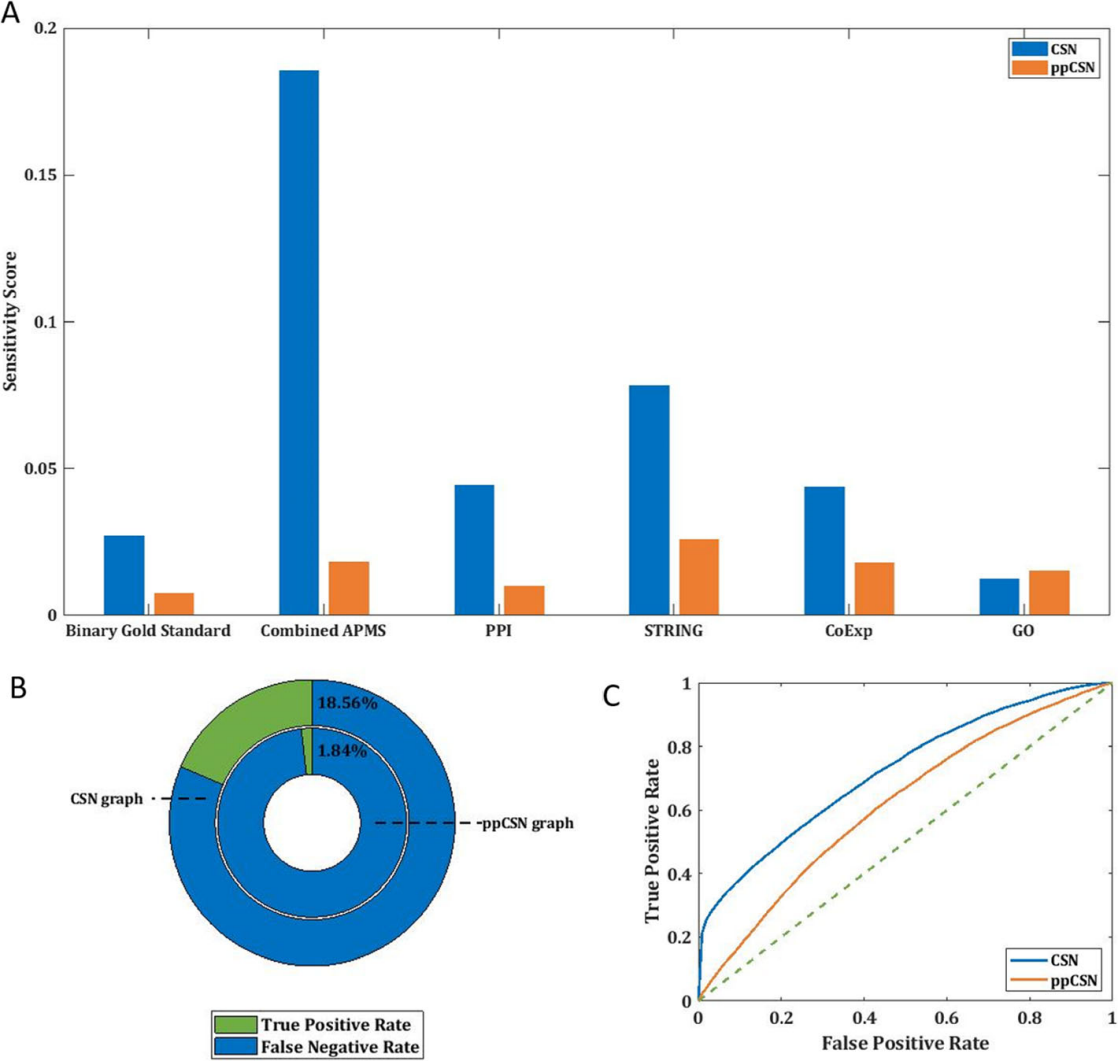
Interactions Prediction. **a** Sensitivity score for interaction predictions for overlap of CSN (blue) or ppCSN (orange) with different genetic networks**. b** The overlap between CSN graph interactions and the co-complex network. In green - the percentage of genes pairs that share a common edge in CSN (outer circle) and ppCSN (the inner circle) and are also known as co-complex interactions among all known co-complex interactions (blue & green). **c** ROC diagram for co-complex interaction prediction. CSN curve in the blue and ppCSN curve in orange

If the sequence-based network that we present here is related to real biological interactions/relations, we expect to see a significant overlap between CSN edges and measured biological interactions between genes or proteins. Thus, we compared CSN edges with experiments-based networks’ interactions (Fig. 3a and Data Source section). The analysis demonstrates that, indeed, the CSN edges overlap with various such measured interactions. The success rate of ppCSN is significantly lower in all cases, suggesting again that the relevant CSN functional information is partly encoded in complex genes’ silent aspects (Fig. 3a and Supplemental Fig. S7).

The co-complex membership associations network [55] is the most compatible network to the CSN among the seven examined networks. CSN predicted 18.55% co-complex interactions, while the ppCSN graph showed a minimal match with the co-complex network interactions (only 1.83% interactions were predicted) (Fig. 3b). The Receiver Operating Characteristic curve [ROC] is based on different edge densities of CSN and ppCSN (Fig. 3c). The ROC curve shows that CSN is more accurate as a predictor of co-complex interactions than ppCSN with AUC (i.e., Area Under Curve) of 0.712 for CSN and AUC 0.608 for ppCSN.

A similar analysis for *E. coli* also demonstrated the advantage of CSN over ppCSN regarding interaction prediction (Fig. S12H).

### CSN is organized based on gene function

**Fig. 4:**
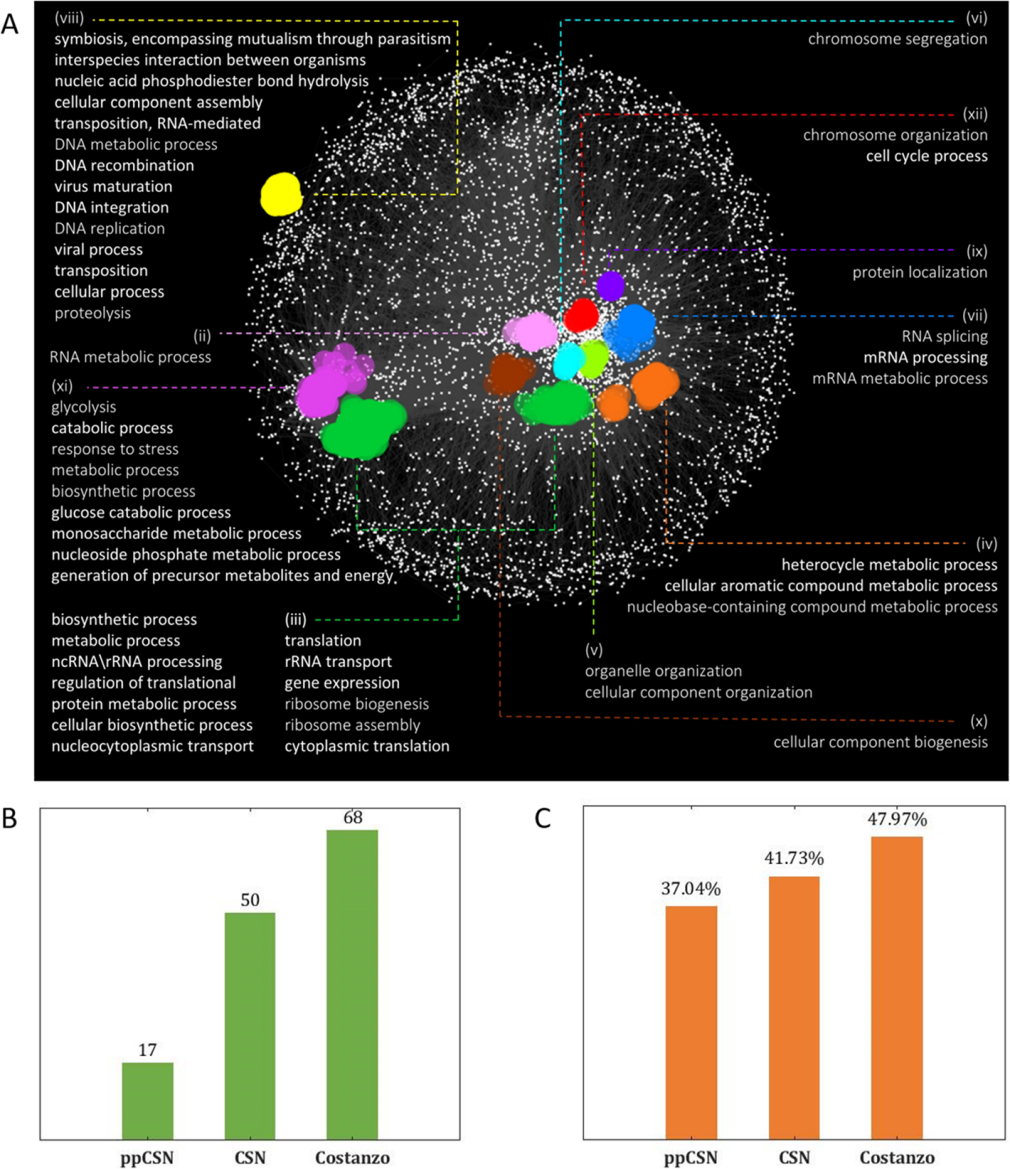
Functional Region Detection. **a** CSN SAFE analysis. SAFE analysis for functional enrichment with the complete *S. cerevisiae* GO map on CSN. Nodes related to each functional domain colored with a specific color and the terms mapped to a domain are listed**. b** The number of terms from the *S. cerevisiae* slim GO map that was detected by SAFE. **c** Sensitivity test. SAFE ability to predict gene functionality with *S. cerevisiae* slim GO map

To show how CSN enables the prediction of gene annotations, we used a network clustering technique called SAFE [56, 57]. SAFE inputs are Gene Ontology [GO] table and a genetic network. The algorithm divides the network’s projection on two dimensions (2D) into ‘regions’ (corresponding to sub-networks) that are enriched with genes that tend to have specific functionality (see more details in the Methods section). Analyzing CSN with SAFE demonstrated that similarly to other biological networks (e.g., genetic interaction networks [25]), the nodes in the CSN are organized by their functionality groups - genes with similar annotations tend to be closer in the CSN graph. Specifically, SAFE identified 132 functional attributes as enriched in 11 specific regions in CSN (see supplementary section, eq. 5, step 5). Those 11 specific regions hold 798 genes. All the genes that were found as scientifically related to the functional attribute in a specific region are color-coded by the functional attribute’s color. Functional attributes with similar genes landscape are grouped as one color (see suplementary eq. 5, step 6, and Fig. 4a).

To make a more accurate assessment of CSN’s nodes tendency to be grouped according to their functionality, we re-analyzed the CSN based on the ‘GO slim’ table. ‘GO slim’ contains 166 main *S. cerevisiae* GO terms, in comparison to the 4373 terms in the ‘full GO’ table. In this analysis, SAFE detected 50 terms in the CSN graph that are enriched within a specific region in the graph 2D projection. Performing false discovery rate [FDR] correction [58] on the SAFE *p*-values output, discovered that CSN has 8574 annotations (term-gene interactions) with significant p-values, and ppCSN have only 1687 annotations with significant p-values. We detected 1396 annotations with p-values < 2.22*10^− 308^ in CSN, while ppCSN have only 17 such significant annotations. As a comparison, a similar analysis of the established genetic interaction network of Costanzo et al. [25], which is based on a vast amount of experiments revealed 68 significant regions with 892 genes from a total of 2838 genes in the network (Fig. 4b). Similar conclusions were obtained for *E. coli*: 9 regions were found in CSN and zero regions for ppCSN (Supplemental Fig. S12I and S12J).

This result reinforces our claim that the information related to the functionality of genes is also encoded in gene’s “silent”/synonymous aspects and not only in its amino-acid content and that our approach can detect some of this information. The CSN has a functional prediction success rate (i.e., Sensitivity Score) of 41.73% for *S. cerevisiae’s* genes and 63.16% for *E. coli’s* genes (Fig. S12K). In other words, 41.73% annotations that SAFE predicted, based on CSN’s genes’ neighborhood enrichment score, are already known to science and appear in the GO table, supporting the suggestion that the CSN is a useful model for gene function inferences. For comparison, Costanzo experimental network success rate is close to CSN’s success rate (47.97%), while the sensitivity score of the ppCSN graph is lower than the CSN sensitivity score (37.04%, Fig. 4c). Other prediction metrics, such as Specificity and Accuracy, are less relevant due to a large number of cases of ‘no associations’ between pairs of genes and annotation (for more details see Supplemental Table S9).

This result shows that the CSN graph is comparable to the Costanzo graph regarding nodes organization with functional regions.

Based on our inferred network, we suggested an approach for predicting novel functional annotations (see details in the Methods section). When we implemented this predictive algorithm on *S. cerevisiae*, *E. coli,* and the Metagenomic sample, we predicted 13,157, 392, and 693 new annotations, respectively (see supplementary Table S13).

### Exploring novel genomes using the CSN algorithm

The CSN algorithm can be applied to any life domains and can help researchers to get an initial indication for the functionality of genes even when annotation data is sparse. To demonstrate this claim, we picked a recently submitted metagenomic sample from the MGnify database [59]. MGYA00382686, a shotgun metagenomics sample from a human gut microbiome (Sapienza Universita’ di Roma, 2019). After preprocessing the metagenomic data, we generated a CSN graph based on all processed reads with predicted CDSs [pCDS].

**Fig. 5:**
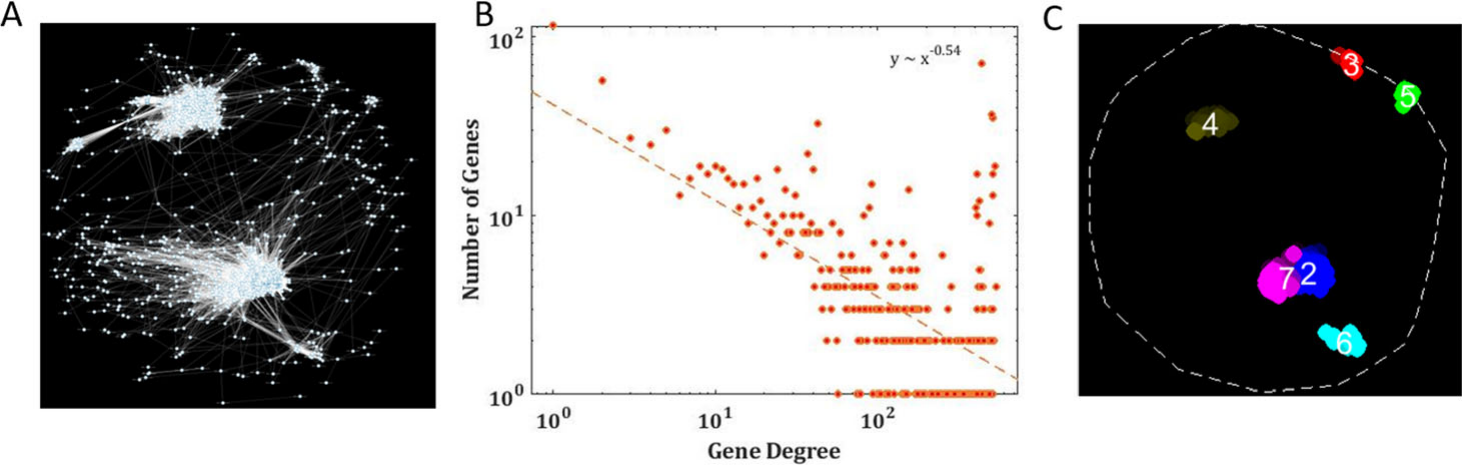
Exploring novel genomes using the CSN algorithm. **a** The Common Substring Network of Metagenomic sample *MGYA00382686*. This network include 1685 nodes (sequences) and 136,996 edges. **b** Node degree distribution. Dot plot describing the distribution of node degrees in a log-log scale for CSN’s nodes. Dashed lines represent the regression lines between degree and number of times it appears in the graph. [Spearman: rho = − 0.512, *p* = 3.89*10^− 23^; Pearson: rho = − 0.204 *p* = 2.05*10^− 4^] **c** Metagenomic sample SAFE analysis. 11 terms are found to be enriched in a certain region: r2 - IPR002932, r3- IPR006860, r4- IPR000209 IPR011991, r5- IPR004358, r6-IPR010930, IPR016156, r7-IPR000795, IPR010559, IPR015883, IPR035684 (see Table S15 for more details about the functions and their definitions)

The MGYA00382686 sample network holds 1685 sequences with 136,996 edges (Fig. 5a). We employed the approach on the coding sequences of the first 99 nucleotides (See supplementary section Determine Sequence Range), inferred from the sample based on nucleotide triplets (see Fig. 1d). Examining CSN’s nodes’ degree distribution in linear fit shows a significant negative correlation between node degree and frequency [Spearman: rho = − 0.512; *p* = 3.89*10^− 23^, Pearson: rho = − 0.204; *p* = 2.05*10^− 4^] (see Fig. 1b).

To test the network ability to predict functionality on a limited annotation data, we created a partial GO table by reading the annotations of the pCDS with the predicted proteins. Then, we ran a SAFE analysis with the partial GO table and obtained the results that appear in Fig. 5c. From 1685 sequences in the sample set, only 905 sequences (53.71%.) had single known functional terms attributed. With this incomplete annotation information, CSN predicted additional annotations for 1132 sequences (67.18% of the sequences). Note that many of the sequences have more than one predicted term (see details in the supplementary Table. S13).

## Discussion

In this article, we present a novel unsupervised approach for understanding the genomes of new organisms. Our new method’s main output is a generated network named the Common Substring Network. The network represents similarities among an organism’s set of genes and enables inferring novel information related to the genes’ functionality and expression levels. This 2D network can also be analyzed with various network analysis tools. When evaluating our reported results it is important to remember that gene interaction networks are very noisy [60]. Specifically, even if you compare two experimental based protein-protein interaction networks, the error rate is 0.179 (see details in the supplementary section Biological Networks Alignments), similar to the one reported here for the CSN. Thus, many features should be combined for such network predictions and the CSN can be one of them.

To demonstrate the approach, we apply it to the model organisms *S. cerevisiae* and *E. coli*’s full gene sets and on a metagenome sample. Results confirm that the CSN method can easily be applied to other organisms from different life domains (See Supplemental Fig. S12 for *E. coli* analysis and Results section for *S. cerevisiae* analysis). We show that the CSN can be used for predicting protein levels (Supplementary Fig. S14) and gene functionality (Supplementary Table S13) and that in many cases the CSN’s performances are comparable to that of biological networks based on expensive and time-consuming biological experiments.

It is essential to emphasize the fact that our measure is not based on conventional homology (e.g., pairwise alignment of complete proteins) and performed well for genes that do not have homologs. It is also based both on information that appears in the coding region and information encoded in the untranslated region.

The CSN considers both the amino acid content of a gene (but also silent aspects encoded in the promoter/UTR and in the coding region). To better understand the information encoded in the CSN, we compared CSN results to ppCSN performances. ppCSN, a network that based on a similar gene set as CSN with a different order of codons or nucleotides that maintains the original network’s encoded proteins sequence, GC content, and codon frequencies. In all cases, CSN significantly outperformed the ppCSN, demonstrating that there is essential information captured by the CSN that does not appear in the amino acid content of proteins or simple genic features such as GC content or codon frequencies.

We believe that our approach or a version of it can be used to study a novel or non-annotated organism when no other experimental information is available except the gene sequences (possibly when combined with additional types of data and algorithms). As we demonstrated here, based on partial information on the gene function (which can be gained, for example, based on the alignment of some of the genes to the orthologs in well-studied model organisms), our CSN can provide additional crucial functional information.

In addition, the CSN can be used to study the complicated gene expression process: we specifically showed that the node degree in the CSN is a feature that can improve the prediction of protein levels when adding it to a more conventional feature like CAI.

This study aimed to demonstrate the general idea behind the CSN. The pipeline described here can be easily improved and generalized in various dimensions and directions. For example, a CSNs that are based on different genome’s parts (e.g., introns, promoters, 3′ UTR) can be inferred separately; these parts can be combined or weighted together to get one network. On the other hand, a researcher can generate a network for each sequence type separately and then compares the different networks. The CSN can also be combined with other types of information or data, including experimental data, to provide accurate predictions, such as gene functionality prediction, expression, and evolution. Also, the CSN can be used for analyzing viruses and micro-organisms separately, and as a community. For example, CSN pipeline input may be metagenomics data, where the genes we examine are related to various organisms, within the same ecological niche, that interact with each other.

Similarly, it may be used to analyze together the genes of a host and its parasite (e.g., a virus) or a set of symbionts to understand the way they co-evolve. Combining genomes from several organisms to one CSN may also reveal evolutionary conservation between sets of genes. One of the ways one can use the CSN algorithm is as follow: First, pick a set of genes from any gene sample. Then, find partial annotation of these genes based, for example, on BLAST or any other method for detecting protein similarity (i.e., comparison to annotated orthologs). Finally, run the algorithm on the gene set (as demonstrated now with the metagenomics example) to generate the CSN. Adding additional predicted functional annotations to the annotation list can be done by using SAFE in the way it presented in the article, as demonstrated with the metagenomics example.

## Methods

### Algorithm description

This subsection gives an in-depth look at the different steps that generate the CSN graph efficiently (see Fig. 1v and supplementary section Running time and Space complexity). For a given set of genomic sequences, the algorithm first calculates the common-chimeraARS scores for all pairs of genomic sequences. Then it summarized the scores in a matrix (score matrix). This matrix is then transformed into a correlation matrix (which based on correlations between rows in the scores matrix). The correlations matrix represents an undirected weighted graph where an edge weight corresponds to a score in the correlation matrix. In this graph, edges with low values (related to low similarity) are removed to make sure that the edges represent high similarity scores and thus are meaningful ones. Next, we greedily added a minimal number of edges to the graph between connected components to make the graph connected. Finally, the sequence-based graph was embedded in a two-dimensional layout and was displayed as the sequence’s Common Substring Network.

### Calculating normalized chimeraARS score

A unique sequence similarity measurement called Chimera Average Repetitive Substring (ChimeraARS) [45] gives a more significant impact to long substrings that are shared between pairs of sequences. It was shown that a version of this measure could be used for ranking different regions of genes according to their expression levels, suggesting that this measure may capture regulatory signals and signals related to the gene’s functionality, which are encoded in different parts of the genes [45].

Genes with similar functionality and similar protein expression are likely to share sub-sequences, and thus will have a higher chimeraARS score. In this study, the chimeraARS score was computed for each pair of sequences, by comparing a target gene *S,* to a reference gene *R*. The algorithm scans the target gene, nucleotide-by-nucleotide if this is a non-translated region, or codon-by-codon if it is a coding region. For each position, it finds the length (*l*_*i*_) of the longest common substrings that starts from position *i* that also appears at the reference gene.

The average length of those substrings is calculated (defined here as *ARS*(S, R)). Next, analysis repeats with *R* as the target gene and *S* as the reference, to get *ARS(R, S).* The final Normalized chimeraARS score for a pair of genes is the average of these two ARS scores (see example in Fig. 1e). This score is computed for all pairs of sequences in the set and arranged as symmetric matrix *M*’.

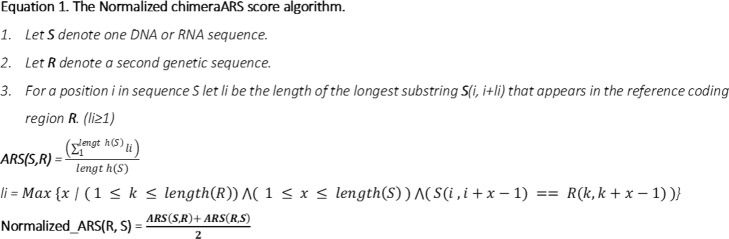



### Algorithm implementation

To calculate the Normalized chimera ARS score for a given pair of sequences efficiently, we created a different Suffix Array [SA] data structure for each sequence [61]. Specifically, for every sequence in the input set, the algorithm creates its unique SA that will be used to calculate the longest common substring between each pair of sequences (Supplemental Fig. S2). One SA is considered the target and the other- the reference SA. For each position in the target sequence (a suffix in the target SA), we searched, using a binary search, the longest matching prefix in the reference SA.

### Combining chimeraARS scores

The normalized ARS scores mentioned above were computed separately for the UTR/promoter region, and the CDS sequence. The UTR/promoter region includes 100 nucleotides and 50 nucleotides before the start codon for *S. cerevisiae and E. coli*, respectively; the CDS sequence includes the first 500 and 250 nucleotides for *S. cerevisiae* and *E. coli* respectively. In the second sequence type, the score is calculated based on codons/nucleotide triplets (Fig. 1c). However, the results reported here are robust to changes in this length threshold (Supplemental section: Determine sequence range and Fig. S1). To combine the two scores, we performed weighted arithmetic mean where the coding regions are weighted five times higher than that of the UTR/promoter weight to reflect the relatively longer region of the coding region that was used:

Aggregated ARS score(R,S) = (|UTR_length| * Normalized ARS scores(R_UTR, S_UTR) + |CDS_length| * Normalized ARS scores(R_CDS, S_CDS))/ (|UTR_length| + |CDS_length|).

The reported results are robust to changes in the relative lengths of these two segments (see Supplemental section: CSN based on coding and regulatory regions separately, and Supplemental Fig. S11). This combined score is computed for all pairs of sequences in the input set and arranged as symmetric matrix *M.*

### From scores matrix to correlation matrix

In the next step of the algorithm, Spearman’s correlation for each pair of rows *x,* y in *M (Aggregated ChimeraARS score matrix)* is computed to generate a correlation-based scoring matrix *Mr* where *Mr(x, y) = rho = Spearman_correlation(x, y).* The algorithm compares rows that represent the sequences’ scores sets. Note that comparing columns would yield the same result due to matrix symmetrically. We used this measure as it compares for each pair of sequences (x,y) the set of normalized chimeraARS scores related to sequence x to the set of normalized chimeraARS scores related to sequence y.

This type of comparison includes more information than just using one normalized ARS scores related to x and y since it considers N relations instead of only one (where N denotes the number of input sequences).

We used Spearman’s rank correlations in this case because we do not expect a linear relationship between pairs of analyzed variables, and we do expect to see monotonic relations (See example in Fig. 1b, Step 3).

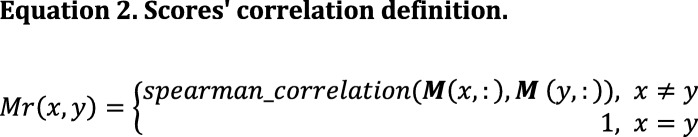



### From Correlation’s matrix to a CSN graph

The matrix *Mr* can be represented as a complete graph with edges representing ‘similarity’ score among pairs of genes; however, such a graph is ‘noisy’ if it includes edges with very low weights. Thus, we filtered edges based on their corresponding correlation scores: Only edges above a minimal weight (i.e., specific correlation) were included. We reported here results related to a threshold of RHO = 0.6 (note that the *p*-value related to all these edges was significant). However, the results are robust to changes in this threshold (Supplemental Fig. S4).

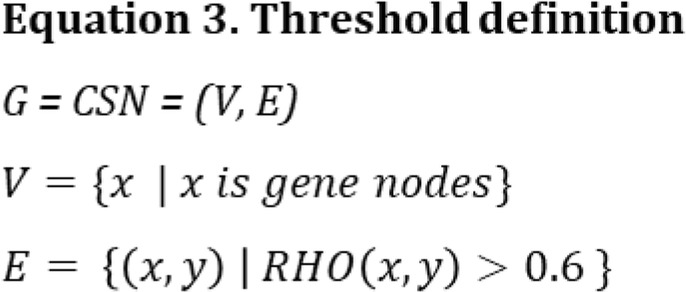



### Determine edge cutoff

In *S. cerevisiae*, the edge weight threshold is 0.6; to keep the graph sparse enough to visualize it properly, In *E. coli***,** the minimum correlation score is set to 0.42 to achieve the same edges density as *S. cerevisiae* CSN (Supplemental Fig. S12F).

### Ensuring graph connectivity

The previous step may generate a disconnected graph that cannot be efficiently dealt with the network embedding algorithm (see next sub-section). Thus, in the next step, we transformed the graphs obtained in the previous step to a connected graph by adding a minimum number of edges that were not included in the initial graph while greedily choosing at each step the maximal additional edge’s weights. This algorithm is a modification of the Kruskal algorithm that connects graph components instead of nodes.

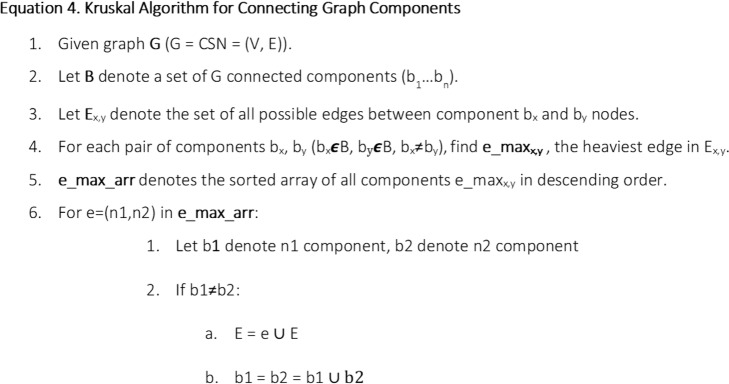



### Network embedding and visualization

Finally, to visualize the graph in 2D, we used Cytoscape software [62]. We used a force-directed layout algorithm as the embedding algorithm for setting the node and edge’s locations in 2D [63]. The force-directed layout algorithm sets the graph topology by force equation where nodes push each other away, but edges between nodes pull them together. The attraction between two nodes is correlated to the weight of the edge between them. This way, nodes with heavier weight (in our case, higher similarity) tend to be physically closer in the graph.

### Randomization and validation

To estimate the importance of silent aspects of the genes on CSN performance, we created a reference network we called ppCSN, which maintains the amino acid sequence of the gene, its GC content, and the codon frequencies but not the exact nucleotide order.

Randomization was done as follows:

(1) To randomize the gene’s non-translated sequences (e.g., promoters and introns), we performed permutations on their nucleotides.

(2) To randomize the gene’s translated sequence (e.g., CDS), while keeping the amino acid chain and genomic codon bias, we rearranged all synonymous codons within and between sequences (Fig. 1d).

(3) Then we used the same pipeline as CSN on this partly-shuffled sequence (Fig. 1b);

(4) we made sure that the number of edges (and nodes) in the CSN and ppCSN was identical by adding edges according to their weights in addition to ppCSN edges.

## Supplementary information

**Additional file 1.** Additional Methods and Results.

**Additional file 2.** Table 13a. The new 13,157 annotations predicted for S. cerevisiae. (B) The new 392 annotations predicted for E. coli. (C) The new 693 annotations predicted for the metagenomic sample.

**Additional file 3.** Table 13b. The new 392 annotations predicted for E. coli. (C) The new 693 annotations predicted for the metagenomic sample.

**Additional file 4.** The new 693 annotations predicted for the metagenomic sample.

## Data Availability

The sources of all the used data appear in the Methods section.

## Abbreviations

SA: Suffix Array
CDS: coding DNA sequence
CSN: Common Substring Network
ppCSN: protein preserving Common Substring Network
SAFE: Systematic functional annotation and visualization
GO: Gene Ontology
PA: Protein Abundance
TPR: True Positive Rate
FPR: False Positive Rate
ROC: Receiver Operating Characteristic
AUC: Area under curve
pCDS: Processed reads with predicted CDS
