# Supplementary material for: CSN: unsupervised approach for inferring biological networks based on the genome alone
Source: BMC Bioinformatics. 2020 May 15;21:190. doi: 10.1186/s12859-020-3479-9 (PMC7227238; doi:10.1186/s12859-020-3479-9)
Supplement: Supplementary file 4 — Additional file 4. The new 693 annotations predicted for the metagenomic sample. [file 12859_2020_3479_MOESM4_ESM.pdf]

Supplementary Information for the paper "CSN: Unsupervised Approach for Inferring Biological Networks based on the Genome Alone"

Maya Galili, Tamir Tuller

Results for *E. coli*'s CSN

We performed the analysis for an additional organism - the *E. coli*. All the results appear in supplemental Fig. S12. Conclusions for *E. coli* are similar to the one reported for *S. cerevisiae* and described in the main text.

Running time and Space complexity:

L = gene (or subsequence) length

N = number of genes

Pre-processing: Suffix Array creation:

Time: O(N*L*log(L)) [creating the Suffix Array O(N*L) + sorting the arrays O(N*L*log(L))

Space: O(N*L)

<u>Step 1: Calculating the Normalized ARS score for a pair of genes:</u>

Time: O(L*log(L)) [binary search to find g1 suffixes in g2 suffix array].

<u>Step 2: Calculating the Normalized ARS score for all pairs:</u>

Time: O(N2*L*log(L))

* Using parallel calculation methods can significantly reduce running time, as the scores can be calculated independently for each gene pair.

<u>Step 3: Compute the correlation matrix</u>

The running time for calculating correlations between all pairs of the matrix rows is O(N3).

Space: O(N2), We can use the same matrix.

<u>Step 4: cut out low-weight edges</u>

Time: O(N2)

Space: O(N2) [We can use the same matrix]

Step 5: connect components

Using Kruskal's algorithm or Prim's algorithm for computing a maximum-weight spanning tree takes O(|E|*log|E|) where |E| is the number of optional edges of step 4 output graph.

Step 6: graph drawing:

The force-directed algorithms, in general, are having a running time of O(N*Log(N)) (Kobourov,SG. Et al., 2012), where N is the number of nodes. In our case, N equals the number of sequences in the input set.

To speed up the process, we can use Graphviz software that is faster as it implements a multilevel force-directed layout algorithm and also capable of handling very large graphs.

Running time in practice:

For example, a PC with Intel® Core™ i7=4790 CPU @ 3.60GHz 3.60 performance will generate a network file for 100 sequences, 250 nucleotides long in ~17.537 seconds

Reference networks

Binary-GS: widely-used Binary gold standard network (Mewes, 2006)

Costanzo 2010: A global genetic interaction network (24)

STRING: functional protein association networks (Szklarczyk et al., 2017)

PPI: protein-protein interaction (Diament et al., 2014)

CoExp: correlation coefficient between the transcription levels of pairs of genes network (Diament and Tuller 2017)

Combined APMS: Co-complex membership associations network [from yeast interactomes database] and co-complex network (Collins et al., 2007)

For the *E. coli* interactions' prediction, we used four networks from the EcoliNet database (Kim et al., 2015)

Integrated: EcoliNet.v1. An integrated network.
Co-Functional Gold Standard: EcoCyc.goldstandardset. Positive gold standard co-functional gene pairs.
Co-Functional GO-BP EcoCyc: EcoliNet.v1.benchmark. Co-functional gene pairs by GO-BP or EcoCyc annotations.
Co-Functional GO-BP: GO-BP.goldstandardset. Co-functional gene pairs by GO-BP annotations.

The GO table we used for the annotation on the *E. coli* map taken from the Gene Ontology Consortium database (Ashburner et al., 2000)

Data Source

As a proof of concept, we chose to construct the CSN on *S. cerevisiae* and *E. coli* genomes due to a large amount of available information regarding their gene interactions, expression level data, gene functionality, and more. As reference networks, we used well-established biological networks to show that the CSN can be competitive to these networks, which are based on experimental data.

Both organisms' protein level data was downloaded from the PaxDB site (Wang et al., 2015). For *S. cerevisiae*, we used the " 4932-WHOLE_ORGANISM-integrated.txt" file published in 2015, and For *E. coli,* the "511145-WHOLE_ORGANISM-integrated.txt" file also published in 2015. From the initial set of 6008 *S. cerevisiae* genes, 91 genes' protein levels were missing from the database and were ignored in the relevant test. Similarly, 223 genes were missing for *E. coli* and hence were ignored.

All *S. cerevisiae* and *E. coli* gene sequences and was download from the NCBI database (O'Leary et al., 2016) on July 30, 2017, in GeneBank's full format. Gene ontology information for the S. cerevisiae slim Gene Ontology [GO] set was downloaded from SGD Project on 6-Nov-2017 (Cherry et al., 2012). The full GO map we used for Fig. 4A was download from the SAFE project (https://github.com/baryshnikova-lab/safe-java). Both GO maps included the three functional ontologies: biological process, molecular function, and cellular component. The metagenome data was extracted from the MGnify site (Alex L. et al., 2017) with *MGYA00382686* as the example sample. This sample is part of Study MGYS00005107 (PRJNA394849); NGS approaches to metagenomics of the Human gut microbiome. Two files contain the sequence pCDS and Predicted CDS with annotation. Another file mapped the annotation ID to its definition. The CDS prediction is performed using FragGenScan on the reads, and InterProScan annotated all predicted proteins.

The additional information for the InterPro entries was mapped by the InterPro database (Mitchell, A. et al., 2014).

Evaluating the CSN based on Coding Region and Region Upstream of it Separately

The sequence range we used for the *S. cerevisiae* and the *E. coli* CSNs in the article is the beginning of the coding region together with the coding region's upstream sequence (See Fig. 1C).

To demonstrate that the sequence range we used in order to infer the CSN, is superior to using only the coding region or using only the sequence upstream of the coding region, we repeated the analyses for each of these two regions separately and compared the performances.

As can be seen in figure S11, the combined network is more informative for PA and interaction prediction. The CSN that based only on the coding region gets a slightly better result for functionality prediction. These results suggest that in general, it is better to work with the combined score and that for function prediction, the upstream region may be omitted.

Determine Sequence Range

We chose to implement our algorithm on genomic sequences with a constant length and with a similar range around the start site of the gene's coding region. Specifically, for *S. cerevisiae*, we picked the end of the gene promoter, starting from 100 nucleotides upstream to transcription initiation site (in the promoter), and ending (up to) 500 nucleotides into the reading frame. These regions tend to hold many gene regulatory signals, and we expect that the gene functionality is related to them (Stepanova et al., 2005; Ren et al., 2000; Shafee et al., 2017). We set a fixed length of sequences to prevent the length of the sequences from affecting the Normalized chimeraARS scores (Fig. 1C).
From 6,008 *S. cerevisiae* genes, we removed 36 genes (Supplemental Fig. S6) with a coding sequence shorter than 120nt. Assuming these are, most likely, hypothetical genes, based on their description.

For *S. cerevisiae*, the CDS sequence length was set to 500 nucleotides, based on the fact that 85% of the analyzed coding regions are longer than 500 nucleotides. The organism's average coding region length is 1485 nucleotides. Moreover, it is known that the region with various regulatory signals is usually near the start codon (figure S8) (Tuller and Zur, 2015).

Coding regions shorter than 500nt were considered with their real length. Coding regions shorter than 120nt were removed from all analyses (Supplementary Table S6).

S. cerevisiae's promoter length was set to 100 nucleotides since with this length, in 96% of genes, the defined promoter sequence will not include the previous gene. The average distance between *S. cerevisiae*'s coding regions is 565 nucleotides (See supplementary fig S3). *E. coli* sequences start 50 nucleotides upstream to the start codon and end 250 nucleotides downstream to it, based on the gene's length and the distribution of the distance between genes (Fig S12C, S12D).

To make sure we are using the right length of regulatory and coding sequences, we run additional experiments that analyze CSNs with different sequence lengths. Results show that CSN, with the coding region of 500 nucleotides and regulatory region of 100 nucleotides, gets the best scores in all measured aspects (Supplementary Fig. S1).

Data exploration of the Metagenomic sample revealed that all sequences in the sample are longer than 100 nucleotides (see Fig. S16 for lengths distribution of the different genes). For some of the sequences, the suggested amino acid sequence is only part of the nucleotide sequence but always longer than 33 amino-acid. As we did with the other networks, we analyzed the first 99 nucleotides of all the sequences.

Data Preprocessing

To compare CSN with a known biological network created by long term laboratory work, we used several genetic networks, accessible online, like Costanzo interaction network (Costanzo et al., 2016) and other interactomes (see Reference networks section). For the interaction prediction analysis, and for the comparison of PA prediction ability of CSN and the reference networks, we kept the interactions between the common set of networks' genes. To match the two networks' densities and sizes, we removed edges from the denser network in order.

Centrality Analysis

To rank the genes by their centrality in the CSN and other networks, we used their corresponding node degree in the CSN. Since centrality metrics tend to correlate (Yan et al., 2009; Rothenberg et al., 1995), we expect similar results with other centrality measures. An example of centrality scores: PageRank (Page,L. et al., 1999) and node degrees are available for the most and least centered genes of *S. cerevisiae*. (Fig. S10)."

Functional Annotation Analysis

Information about genes known as functional attributes (terms) was taken from the *Gene Ontology DataBase (*Ashburner et al., 2000). We analyzed both the full lists of 4,373 GO terms and the ' GO slim' set, a subset of 166 GO terms. We considered the Biological Process, Molecular Function, and Cellular Component ontologies. In most cases, we focused on the GO slim set.

The SAFE Algorithm

This annotation method aimed at finding "regions" enriched with specific functional annotation (or other characteristics) in a biological network that undergo layout in a 2D, where similar nodes tend to be closer. The network is embedded in 2D based on force-directed layout, where nodes are placed by their edge's weights.

First, for each node in the graph, the algorithm defines a set of nodes, with distance (radius) smaller than a certain threshold as the node's neighborhood. From a radius range of 0-100, the default value was 0.5.

Based on a GO information (a table with the functional annotations of all nodes/genes), the algorithm calculates for each gene g and GO term n a hypergeometric p-value. Suppose that there are x genes in the neighborhood of g with the term n. A hypergeometric p-value is computed based on the probability of getting $\geq x$ genes with the term n, in a neighborhood with the same node size as the g neighborhood, that are related to the term n under uniform probability. If enough genes are found as significantly related to the same term and are clustered close to one another in the plotted network, they will be colored with the same color on the output map (see, for example, Fig. 4A (Baryshnikova, 2018; Baryshnikova, 2016); see Equation 5.

For the metagenomic sample, because the graph is small and there are some very low weights, we ran SAFE with one modification; the neighborhood radius was set to 10.

Validating SAFE Results

We used the GO matrix and the SAFE output p-value matrix to calculate a Sensitivity Score for each term in the GO-slim set. The Sensitivity Score (Powers, 2011) is the percentage of genes that are significantly related to a term based on our prediction and also known to be related to the term based on the GO table.

Validating Interaction Prediction

After matching our computational network (CSN/ppCSN) with the reference network densities and sizes, we used the two connectivity matrices to calculate a Sensitivity Score for each of the computational networks and each interaction type. In this case, the Sensitivity Score (Powers, 2011) is the percentage of interactions that appear in our network CSN/ppCSN, which are also known as real interaction based on the reference network. We also calculated interaction prediction hypergeometric p-value, which is the probability of getting $\geq x$ interactions\edges in CSN, from m optional interactions, that are also known as real biological interactions from type t, under uniform probability (see Supplemental Fig. S7).

ROC Diagram

Network's Co-Complex interaction prediction True Positive Rate [TPR] is the percentage of CSN/ppCSN edges that also appear in the co-complex network. The False Positive Rate [FPR] is the percentage of CSN/ppCSN edges that are not part of the co-complex network.

The ROC diagram (Fig. 3C) was generated as follow:

A set of graphs with densities from 0 to 1 (a fully connected graph) was generated by adding edges to the CSN nodes. Next, we calculate the Co-Complex interaction prediction TPR and FPR, for each different density network.

Biological Networks Alignments

Biological networks are not fully aligned, even when related to the same organism and gene set.

Here we run a short experiment that compares the interactions alignment between 2 known biological networks that we used in the interaction prediction section: STRING and Combined APMS networks.

Number of edges in each network: 8766

Number of common edges between networks: 1569

Sensitivity score: 0.179

Supplementary figures

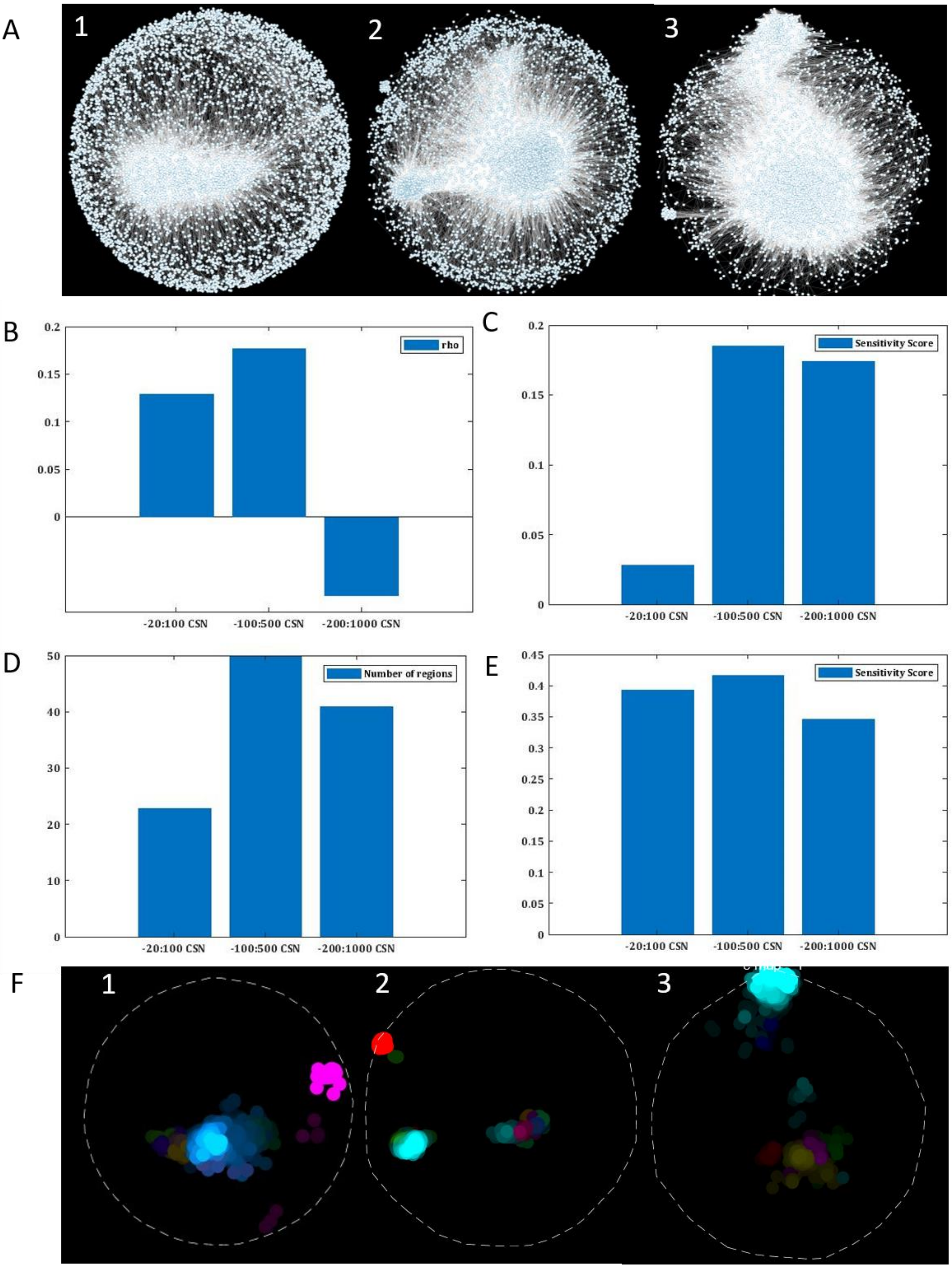

Supplementary figure 1. Analysis of CSNs with different sequence lengths. **(A)** CSN graphs for each sequence length (1) 20:100 (2) 100:500 (3) 200:1000 **(B)** Protein level prediction. PA prediction results **(C)** Interactions Prediction. interaction prediction results. **(D)** Functionality prediction – Sensitivity. functionality prediction sensitivity results. **(E)** Functionality prediction - Functional

Regions. functionality prediction – number of functional regions. **(F)** SAFE result maps. full GO SAFE result maps (1) 20:100 (2) 100:500 (3) 200:1000.

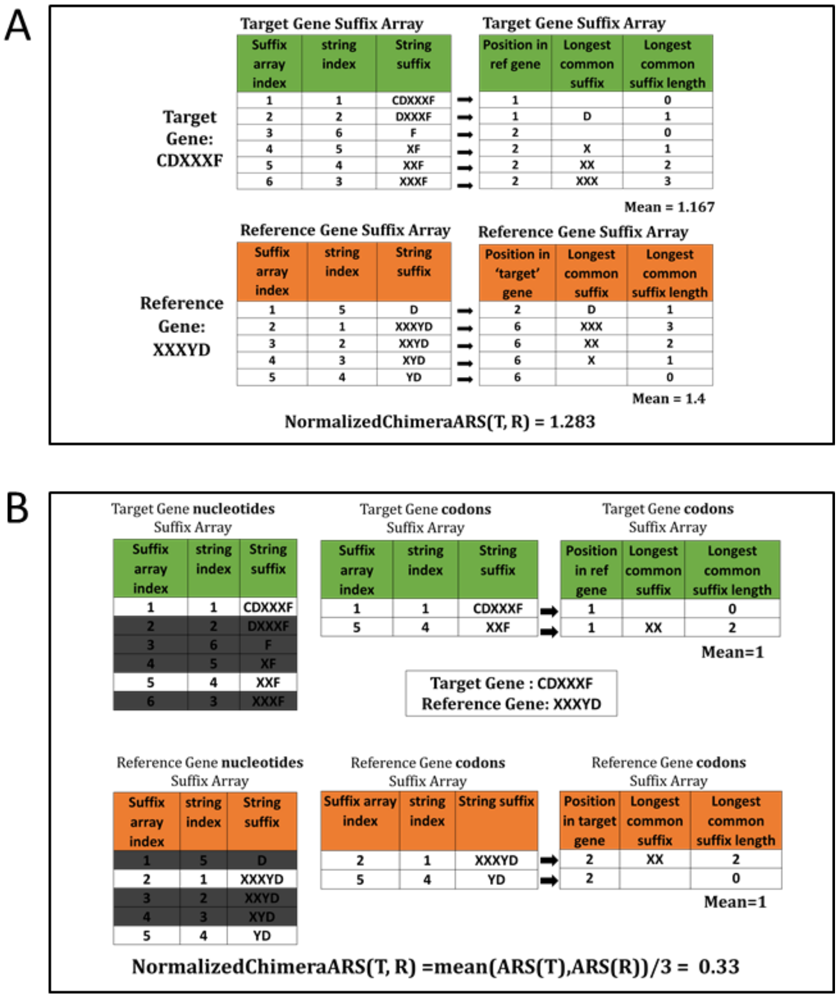

Supplementary figure 2. Normalized chimeraARS Algorithm Implementation. (A) Implementation for the untranslated region: after creating suffix arrays for all sequences, the Normalized chimeraARS score calculated for each pair of SAs. (B) Implementation for the translated region. After creating suffix arrays for all sequences, where only entries that start at codon position(Shendure et al., 2017; Cunha et al., 2016) are left, the Normalized chimeraARS score calculated for each pair of SAs. The final scores are divided by 3.

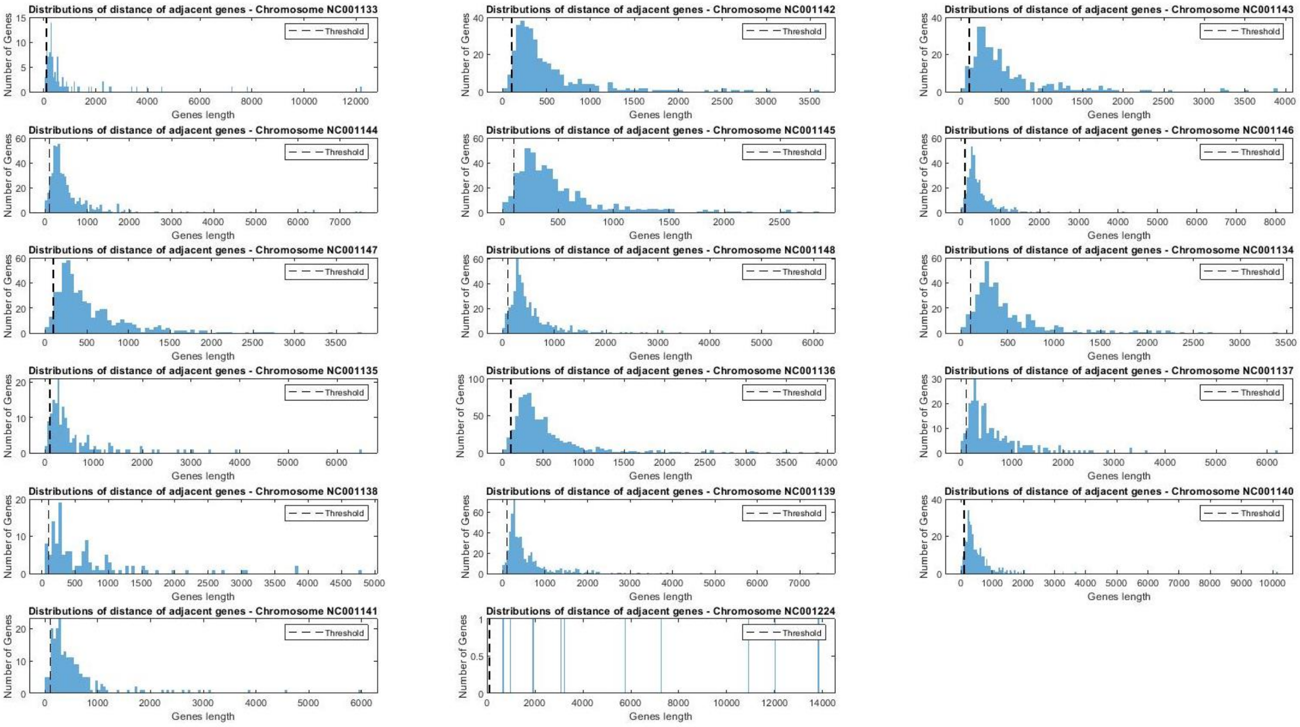

Supplementary figure 3. Yeast promoter's length. Most promoters calculated (95%) are not overlapping with their processor gene if their length set to 100nt.

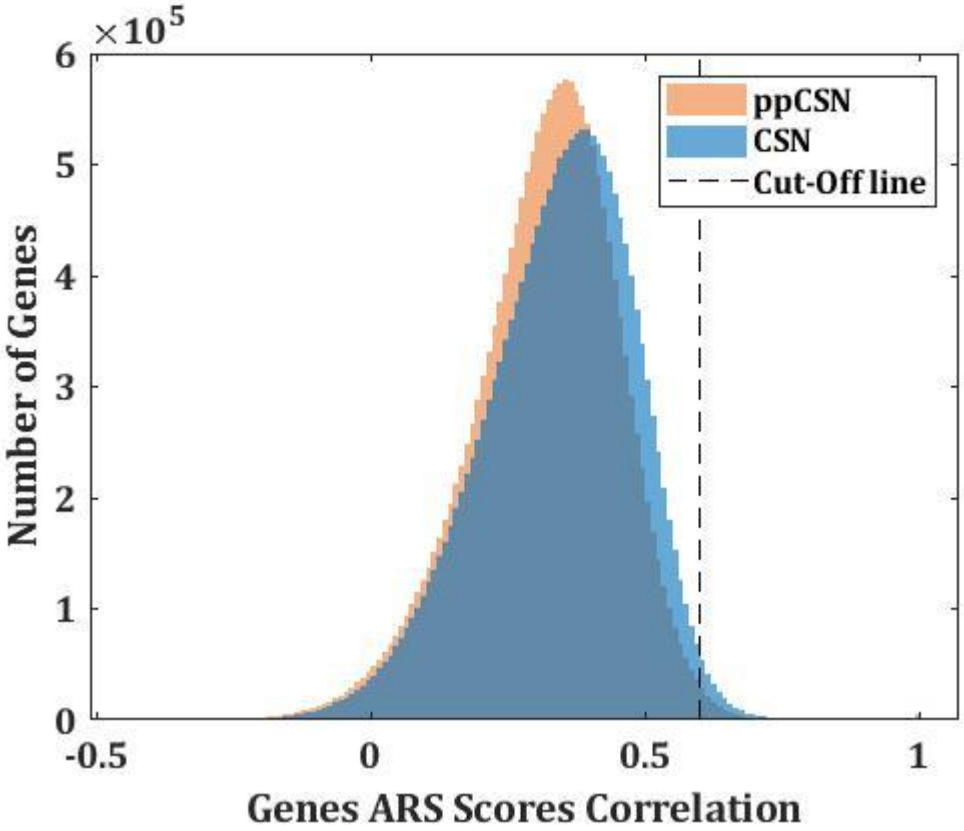

Supplementary figure 4. Scores Correlation Distribution. Comparative analysis of scores correlation distribution between the two sequences sets – the CSN sequences (blue) and protein preserving 'randomized' sequences (orange). Only edges with scores above the dashed line added to the graphs. The CSN matrix scores mean value: 0.341. ppCSN matrix scores mean value: 0.316, and the difference between the two distribution is very significant (Wilcoxon signed-rank test $p < 2.22*10^{-308}$).

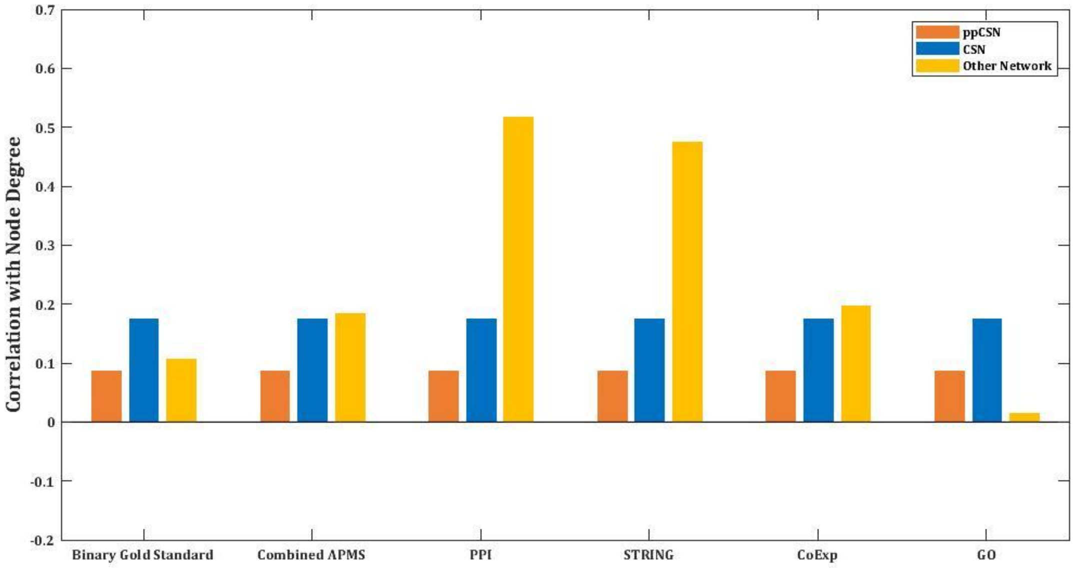

Supplementary figure 5. The correlation between PA and node degree of various *S. cerevisiae* biological networks. The figure includes the correlation between node degree of the CSN (blue), node degree ppCSN (orange), and node degree of other biological networks (see Reference networks in yellow; definitions are in the supplementary section) with PA. In the case reported here, there are no constraints on the number of edges and the number of networks' nodes, as in Fig. 2D.

PROTEIN_ID	ORF_NAME	PRODUCT	PA
NP_878040.1	YAL037C-A	hypothetical protein	0.1280
NP_878042.1	YAR035C-A	hypothetical protein	NaN
NP_878101.1	YJL136W-A	hypothetical protein	NaN
NP_878104.1	YJL077W-B	hypothetical protein	NaN
NP_001257682.1	YJL077W-A	hypothetical protein	NaN
NP_878108.1	YJR151W-A	hypothetical protein	NaN
NP_878129.1	YLR264C-A	hypothetical protein	NaN
NP_076904.1	YML007C-A	hypothetical protein	14.2000
NP_878144.1	YMR182W-A	hypothetical protein	NaN
NP_878147.1	YMR242W-A	hypothetical protein	0.5840
NP_878149.1	YMR272W-B	hypothetical protein	NaN
NP_878150.1	YMR315W-A	hypothetical protein	NaN
NP_014254.1	YNL145W	mating pheromone a	1.1100
NP_878159.3	YNR075C-A	hypothetical protein	1.2200
NP_878164.1	YOL038C-A	hypothetical protein	0.2700
NP_014946.1	YOR302W	hypothetical protein	48.5000
NP_878180.1	YPL152W-A	hypothetical protein	NaN
NP_878184.1	YPR159C-A	hypothetical protein	NaN
NP_878044.1	YBL071C-B	hypothetical protein	NaN
NP_878052.3	YBR196C-B	hypothetical protein	0.9080
NP_878054.1	YBR221W-A	hypothetical protein	0.1590
NP_001291943.1	YCL054W-A	Rdt1p	NaN
NP_010049.1	YDL232W	olichyl-diphosphooligosaccharide--protein glycotransferase OST4	117
NP_010097.1	YDL184C	ribosomal 60S subunit protein L41A	609
NP_010148.1	YDL133C-A	ribosomal 60S subunit protein L41B	371
NP_010749.3	YDR461W	mating pheromone a	284
NP_878066.3	YDR524W-C	hypothetical protein	NaN
NP_061492.1	YER053C-A	hypothetical protein	65.2000
NP_011065.1	YER138W-A	hypothetical protein	NaN
NP_076889.1	YFR012W-A	hypothetical protein	1.0600
NP_878075.3	YGL007C-A	hypothetical protein	NaN
NP_878076.1	YGL006W-A	hypothetical protein	NaN
NP_878081.3	YGR174W-A	hypothetical protein	NaN
NP_878082.1	YGR204C-A	hypothetical protein	NaN
NP_878085.1	YHL015W-A	hypothetical protein	0.4370
NP_878087.1	YHR022C-A	hypothetical protein	NaN

Supplementary figure 6. The list of excluded genes. Details of 36 *S. cerevisiae* Open Reading Frames (ORFs) excluded from its 6,008 available ORFs due to their short sequence (less than 120 Nucleotide). Such a short sequence may imply that they are only hypothetical. The results when including these genes are similar to the ones reported.

ourNetName	otherNetName	Sensitivity	Specificity	Accuracy	hiperGeoScore	CommonNodes
CSN	Binary_Gold_...	0.3548	0.9119	0.9081	1.8256e-12	164
ppCSN	Binary_Gold_...	0.1837	0.9500	0.9484	6.8675e-04	220
CSN	Combined_AP...	0.5537	0.9444	0.9356	0	509
ppCSN	Combined_AP...	0.1206	0.9539	0.9480	1.8111e-27	611
CSN	PPI	0.1031	0.9815	0.9741	0	3395
ppCSN	PPI	0.0308	0.9732	0.9650	1.9623e-06	2893
CSN	STRING	0.0784	0.9871	0.9743	0	5800
ppCSN	STRING	0.0283	0.9847	0.9707	0	5464
CSN	CoExp	0.0439	0.9856	0.9666	0	4827
ppCSN	CoExp	0.0181	0.9851	0.9655	1.6180e-34	4822
CSN	GO	0.0124	0.9825	0.9376	1.0000	3181
ppCSN	GO	0.0153	0.9837	0.9388	0.9999	3180

Supplementary figure 7. The Interaction Prediction Metrics table. Sensitivity, Specificity, Accuracy scores, and hypergeometric p-value for our CSN network vs. other biological networks. Some of the results are summarized in Fig. 3A.

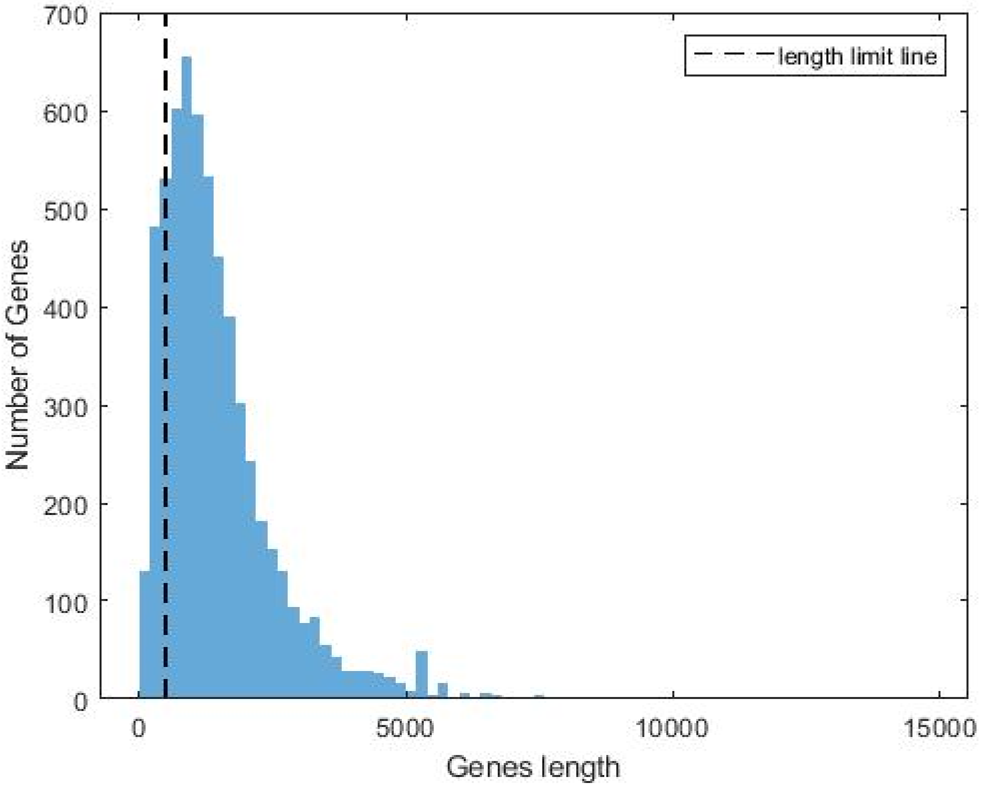

Supplementary figure 8. Yeast genes length distribution. CDS sequence length set to 500nt based on the fact that 85% of the analyzed coding regions are longer than 500nt.

NetName	sigTermsSize	Sensitivity	Specificity	Accuracy	FNR	FPR	errRate3	hiperGeoScore	oncesInFirstMat	oncesInSecMat	possibleOnces
ppCSN_slimGO_for_100_500	17	0.3704	0.9842	0.9607	0.6296	0.0158	0.0393	0	29122	37828	991352
CSN_slimGO_for_100_500	50	0.4173	0.9714	0.9502	0.5827	0.0286	0.0498	0	43060	37828	991352
Costanzo_slimGO	68	0.4797	0.9719	0.9522	0.5203	0.0281	0.0478	0	21766	18850	471108

Supplementary figure 9. Functional region detection table.

A

GeneName	GeneProduct	PA	NodeDegree	PageRank
YAL067W-A	hypothetical protein	0.0750	1	9.4442e-05
YAL065C	hypothetical protein	1.1500	1	5.8938e-05
YAL064W-B	hypothetical protein	1.3100	1	9.5251e-05
YAL064C-A	Tda8p	1.4100	1	5.8756e-05
YAL064W	hypothetical protein	0.9190	1	6.6663e-05
YAL063C-A	hypothetical protein	0.1000	1	8.1882e-05
YAL061W	putative dehydrogenase BDH2	34.4000	1	2.7206e-05
YAL058W	calnexin	43.3000	1	2.6348e-05
YAL056W	Gpb2p	2.6400	1	2.6398e-05
YAL054C	acetate--CoA ligase 1	89.4000	1	2.6173e-05

B

GeneName	GeneProduct	PA	NodeDegree	PageRank
YLR182W	transcriptional regulator SWI6	41.1000	784	0.0011
YDL036C	pseudouridine synthase PUS9	8.6600	784	0.0011
YLR306W	NEDD8-conjugating protein UBC12	2.9800	787	0.0012
YNR004W	Swm2p	16.8000	792	0.0011
YLR166C	exocyst subunit SEC10	38.3000	817	0.0013
YCL067C	homeodomain mating type protein alpha2	0.8720	833	0.0012
YCR039C	homeodomain mating type protein alpha2	1.2400	833	0.0012
YOR257W	centrin	4.9600	834	0.0012
YML071C	Golgi transport complex subunit COG8	70.5000	863	0.0013
YDR484W	Vps52p	35.6000	895	0.0013

Supplementary figure 10. Description of most and least centered genes in CSN (A) 15 lowest degree genes. (B) 15 highest degree genes.

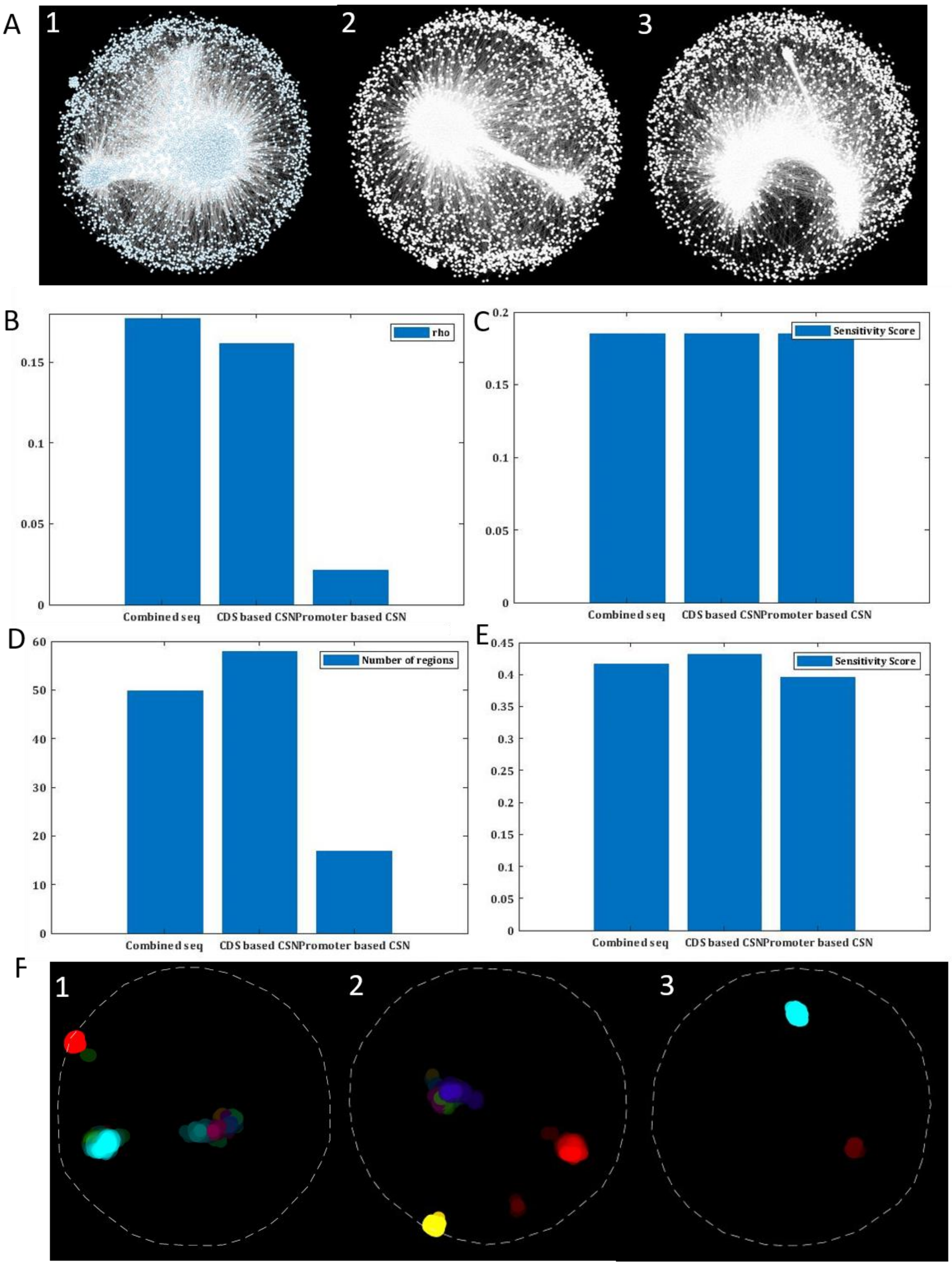

Supplementary figure 11. Comparing CSN based only on CDS and CSN based only on promoters. (A) CSN graphs for each sequence type (1) Combined sequences (2) CDS based sequences (3) promoters-based sequences.

(B) Protein level prediction results. **(C)** interaction prediction results. **(D)** functionality prediction sensitivity results. **(E)** functionality prediction – number of functional regions**. (F)** SAFE result maps. Full GO SAFE result maps (1) Combined sequences (2) CDS based sequences (3) promoters-based sequences.

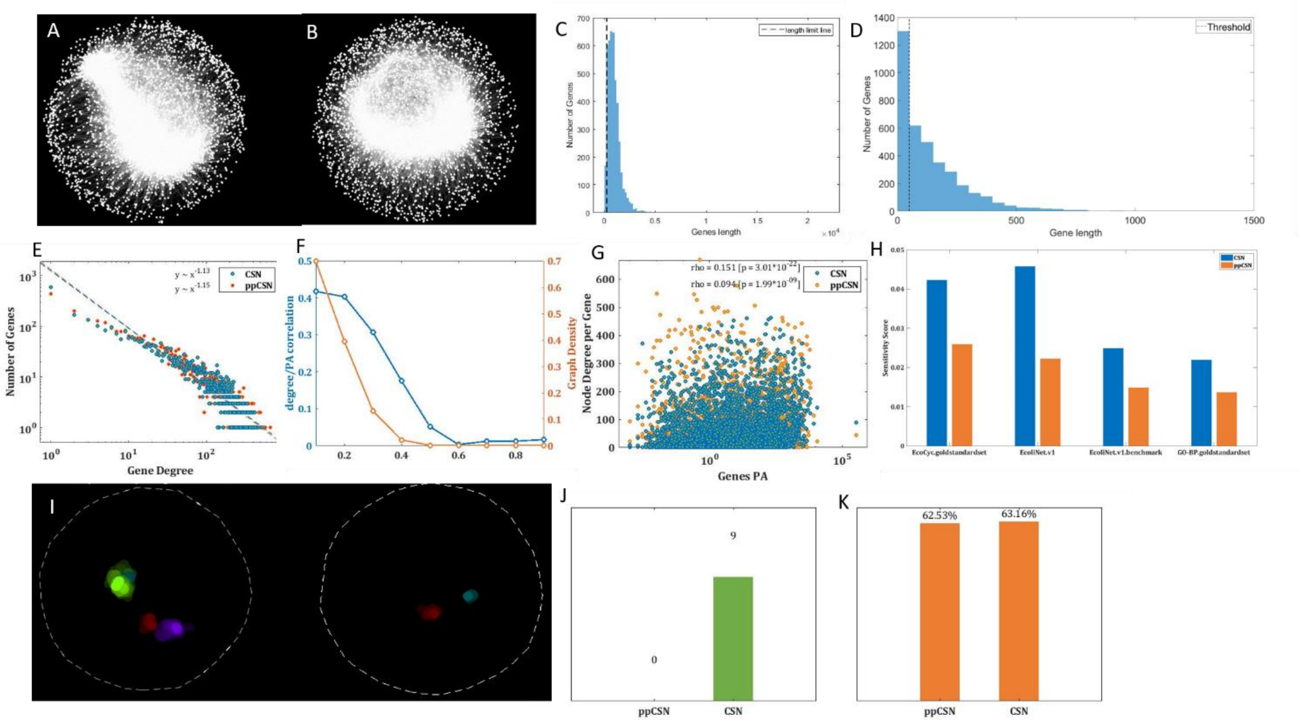

Supplementary figure 12. *E. coli* Common Substring Network Analysis

(A) *E. coli*'s CSN. *E. coli* Common substring network (CSN) contains 4,256 genes and 127,349 edges. **(B)** *E. coli's* ppCSN. **(C)** *E. coli* genes distance distribution. The genetic sequence used here included 50 nucleotides upstream to start codon and up to 250 nucleotides downstream to the gene start codon. **(D)** Degrees' Distribution of genomic distance between genes. **(E)** Degrees' distribution. Pearson correlation between node degree and node degree frequency: CSN R=-0.363 P=3.41*10^{-12}; ppCSN R=-0.408 P=3.64*10^{-16}; Spearman correlation: CSN R=-0.913 P=2.26*10^{-135}; ppCSN R=-0.897 P=1.65*10^{-131}. **(F)** PA prediction for different graph densities. The graph includes the correlation between genes-node degrees and PA per different graph densities (Orange Y-axis) for different edge weight X- thresholds (X-axis). The threshold was eventually is set to 0.42 with a lower degree/PA correlation than optimal (blue Y-axis). **(G)** Gene node degrees vs. PA. Comparing genes degrees and PA. [correlations: CSN R=0.151 P=3.01*10^{-22}; ppCSN R=0.094 P=1.99*10^{-09}] **(H)** Sensitivity score for interactions overlap of CSN (blue) or ppCSN (orange) with different genetic networks. **(I)** *E. coli* CSN SAFE map (left) and ppCSN map (right). Functional regions colored by their terms. **(j)** Regions' number. The number of terms from the *S. cerevisiae* slim GO map that was detected by SAFE. **(k)** The sensitivity score. SAFE ability to detect gene functionality with *S. cerevisiae* slim GO map.

Supplementary Table 13. Predictions of novel functional annotations that currently do not appear in the organisms' Gene Ontology [GO]. The lists contain annotations that are significant after FDR correction. **(A)** The new 13,157 annotations predicted for *S. cerevisiae*. **(B)** The new 392 annotations predicted *for E. coli.* **(C)** The new 693 annotations predicted for the metagenomic sample.

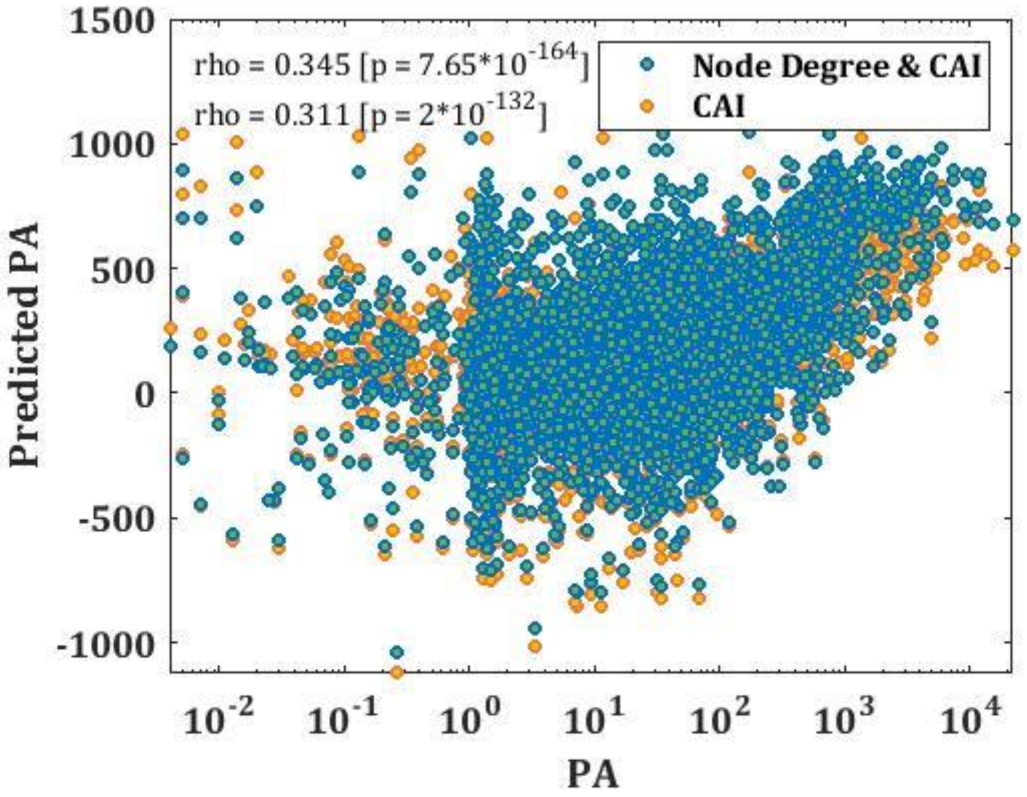

Supplementary Figure 14. Prediction of PA based on CAI and CSN's node degree. The figure includes dot plots of PA vs. a CAI linear regressor (orange) vs. a linear based on CAI and CSN gene's degree (blue).

InterPro ID	Description
IPR002932	Glutamate synthase domain
IPR006860	FecR protein
IPR000209	Peptidase S8/S53 domain
IPR011991	ArsR-like helix-turn-helix domain
IPR004358	Signal transduction histidine kinase-related protein, C-terminal
IPR010930	Flagellar basal-body/hook protein, C-terminal domain
IPR016156	FAD/NAD-linked reductase, dimerisation domain superfamily
IPR000795	Transcription factor, GTP-binding domain
IPR010559	Signal transduction histidine kinase, internal region
IPR015883	Glycoside hydrolase family 20, catalytic domain
IPR035684	Arginyl-tRNA synthetase, catalytic core domain

Supplementary figure 15. Description of 11 InterPro families detected as enriched in a specific region by the SAFE algorithm in the metagenome CSN.

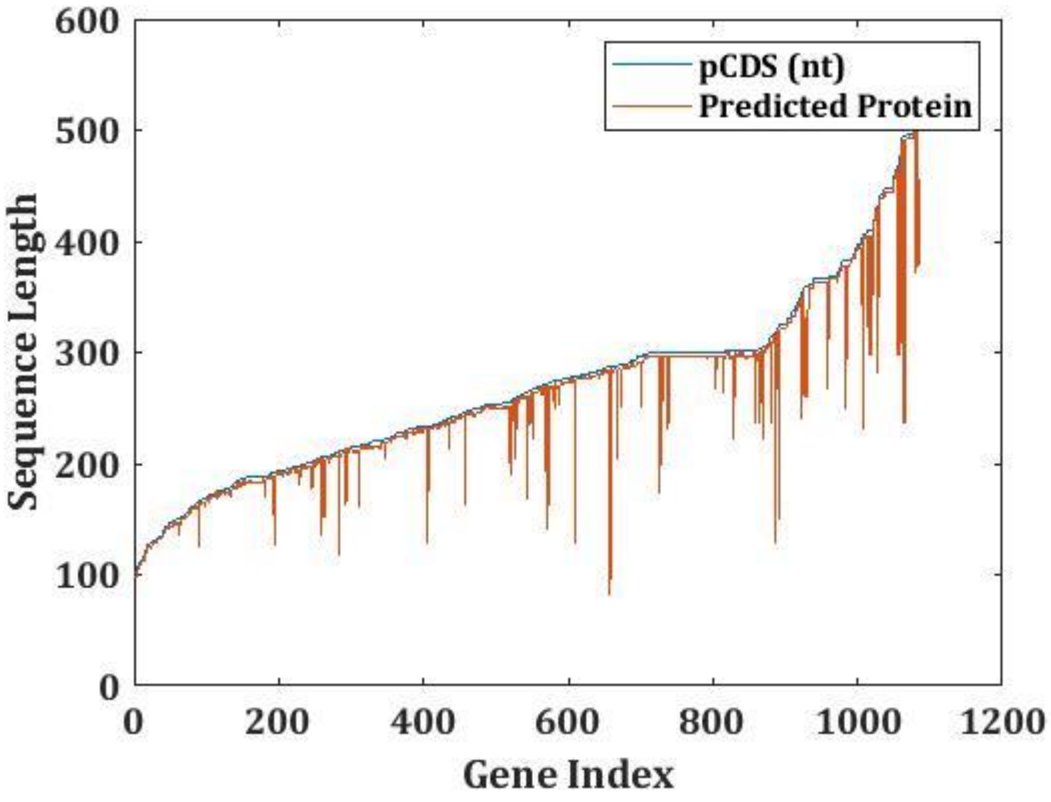

Supplementary figure 16. Lengths of sample nucleotide sequence versus the equivalent predicted amino acid sequence (number of amino acids times three).

	CSN	ppCSN 1 (article reference)	ppCSN 2	ppCSN 3
**Degree vs. PA**	CSN R=0.151 P=3.01*10^{-22}	ppCSN R=0.094 P=1.99*10^{-09}	rho=0.080 p=2.83*10^{-07}	rho=0.090 p=8.69*10^{-09}
**Interaction prediction with 'integrated'**	4.5%	2.1%	2.218%	2.311%
**Number of Functional regions**	9	0	0	1
**Functional detection sensitivity**	63.16%	62.53%	62.38%	61.77%

Supplementary figure 17. Randomization Rubostnase. Repeating the randomization a few times show that the results are similar.

Equation 5. The SAFE method

<u>*Inputs*</u>*: network N, GO table G, and a cutoff distance r.*

1. Apply the force-directed layout algorithm on network N.
2. For every node vi in network G, define a local neighborhood – a set of nodes, such that each node, vj, in the set is with limited Euclidean distance r to the examined node on the network map.

$$C(vi, vj) = \sqrt{(x_i - x_j)^2 + (y_i - y_j)^2} \leq r,$$

$$(x_i, y_i), (x_j, y_j) \text{ are the coordinates of the nodes vi, vj respectively.}$$

- $(x_i, y_i), (x_j, y_j)$ are the coordinates of the nodes vi, vj respectively.

- By default, r equals to the 0.5th-percentile of all pair-wise node distances in the network.
- Radius (denoted here as r) - is the distance threshold that defines the size of the neighborhood. All genes that hold Euclidean distance from the examined node smaller than r on the network map will be considered in its neighborhood.

3. Calculate the enrichment of the node's neighborhoods for each functional attribute

$$\text{For term t and node v: } P(v,t) = 1 - \sum_{k=0}^{S} \frac{\binom{F}{S}\binom{N-F}{U-k}}{\binom{N}{U}}$$

- Where N is the total number of nodes in the network, F is the number of network nodes that are known to be related to the functional attribute t, U is the number of nodes in the neighborhood of node v, and S is the number of nodes in the neighborhood of node v that known as related to functional attribute (term) t.

4. Determine if node v neighborhood is significantly enriched for term t if P(v,t)<=0.05.
5. Define functional attribute as region-specific, if at least 65% of its significantly enriched neighborhoods are within a distance of 2r from each other, where r is the distance threshold that defines the size of the radius of the neighborhood (see step 2).
6. **Enrichment landscape** is defined as the enrichment scores' vector for a specific functional attribute per all network's nodes.
7. Group functional attributes into functional domains based on the similarity (correlation) of their enrichment landscapes.
8. **The functional domain** is defined as the functional attributes' groups with enrichment landscapes' similarity.
9. The Algorithm assigned the same color to all functional attribute in the same domain.

<u>*Outputs:*</u> *(1) composite functional attribute map of the network. (2) p-values matrix of the gene-term enrichment scores. (3) list of functional domains.*

References

1. Mewes HW. MIPS: analysis and annotation of proteins from whole genomes in 2005. Nucleic Acids Res. 2006;34:169–72.

2. Diament A, et al. Three-dimensional eukaryotic genomic organization is strongly correlated with codon usage expression and function. Nat Commun. 2014;5:5876.

3. Diament A, Tuller T. Tracking the evolution of 3D gene organization demonstrates its connection to phenotypic divergence. Nucleic Acids Res. 2017;45(8):4330–43.

4. Kim H, et al. EcoliNet: a database of cofunctional gene network for Escherichia coli. Database. 2015.

5. Cherry JM, et al. Saccharomyces genome database: the genomics resource of budding yeast. Nucleic Acids Res. 2012;40:700–5.

6. Stepanova M, et al. Comparative analysis of relative occurrence of transcription factor binding sites in vertebrate genomes and gene promoter areas. Bioinformatics. 2005;21:1789–96.

7. Ren B, et al. Genome-wide location and function of DNA binding proteins. Science. 2000;290:2306–9.

8. Shafee T. et al. (2017) Eukaryotic and prokaryotic gene structure. WikiJournal Med, 4, 0–3.

9. Yan E, Ding Y. Applying centrality measures to impact analysis: a coauthorship network analysis. The American Society for Information. Sci Technol. 2009;60:2107–18.

10. Rothenberg RB, et al. Choosing a centrality measure: epidemiologic correlates in the Colorado Springs study of social networks. Soc Network. 1995;17:273–97.

11. Ashburner M, et al. Gene ontology: tool for the unification of biology. Nat Genet. 2000;25:25.

12. Power D. M. (2011) Evaluation: from precision, recall and F-measure to ROC, informedness, markedness and correlation.

13. Wang M, et al. Version 4.0 of PaxDb: protein abundance data, integrated across model organisms, tissues, and cell-lines. Proteomics. 2015;15:3163–8.

14. Mitchell A, et al. The InterPro protein families database: the classification resource after 15 years. Nucleic Acids Res. 2014;43:213–21.
